# Assessment of Upper Extremity Function in Multiple Sclerosis: Feasibility of a Digital Pinching Test

**DOI:** 10.2196/46521

**Published:** 2023-10-02

**Authors:** Jennifer S Graves, Marcin Elantkowski, Yan-Ping Zhang, Frank Dondelinger, Florian Lipsmeier, Corrado Bernasconi, Xavier Montalban, Luciana Midaglia, Michael Lindemann

**Affiliations:** 1 Department of Neurosciences University of California San Diego, CA United States; 2 F. Hoffmann-La Roche Ltd Basel Switzerland; 3 Department of Neurology-Neuroimmunology Multiple Sclerosis Centre of Catalonia, Vall d’Hebron University Hospital Barcelona Spain; 4 Department of Medicine Autonomous University of Barcelona Barcelona Spain

**Keywords:** multiple sclerosis, smartphone sensor, digital health technology tools, upper extremity function, hand-motor dexterity

## Abstract

**Background:**

The development of touchscreen-based assessments of upper extremity function could benefit people with multiple sclerosis (MS) by allowing convenient, quantitative assessment of their condition. The Pinching Test forms a part of the Floodlight smartphone app (F. Hoffmann-La Roche Ltd, Basel, Switzerland) for people with MS and was designed to capture upper extremity function.

**Objective:**

This study aimed to evaluate the Pinching Test as a tool for remotely assessing upper extremity function in people with MS.

**Methods:**

Using data from the 24-week, prospective feasibility study investigating the Floodlight Proof-of-Concept app for remotely assessing MS, we examined 13 pinching, 11 inertial measurement unit (IMU)–based, and 13 fatigability features of the Pinching Test. We assessed the test-retest reliability using intraclass correlation coefficients [second model, first type; ICC(2,1)], age- and sex-adjusted cross-sectional Spearman rank correlation, and known-groups validity (data aggregation: median [all features], SD [fatigability features]).

**Results:**

We evaluated data from 67 people with MS (mean Expanded Disability Status Scale [EDSS]: 2.4 [SD 1.4]) and 18 healthy controls. In this cohort of early MS, pinching features were reliable [ICC(2,1)=0.54-0.81]; correlated with standard clinical assessments, including the Nine-Hole Peg Test (9HPT) (|*r*|=0.26-0.54; 10/13 features), EDSS (|*r*|=0.25-0.36; 7/13 features), and the arm items of the 29-item Multiple Sclerosis Impact Scale (MSIS-29) (|*r*|=0.31-0.52; 7/13 features); and differentiated people with MS-Normal from people with MS-Abnormal (area under the curve: 0.68-0.78; 8/13 features). IMU-based features showed similar test-retest reliability [ICC(2,1)=0.47-0.84] but showed little correlations with standard clinical assessments. In contrast, fatigability features (SD aggregation) correlated with 9HPT time (|*r*|=0.26-0.61; 10/13 features), EDSS (|*r*|=0.26-0.41; 8/13 features), and MSIS-29 arm items (|*r*|=0.32-0.46; 7/13 features).

**Conclusions:**

The Pinching Test provides a remote, objective, and granular assessment of upper extremity function in people with MS that can potentially complement standard clinical evaluation. Future studies will validate it in more advanced MS.

**Trial Registration:**

ClinicalTrials.gov NCT02952911; https://clinicaltrials.gov/study/NCT02952911

## Introduction

Multiple sclerosis (MS) is a chronic autoimmune, demyelinating, neurological disease [1]. Impaired upper extremity function commonly affects people with MS, with approximately 60%-76% of them experiencing or showing signs of it during their disease course [2-4]. The impairment can hinder their daily activities and reduce their quality of life (QoL) [5]. Given such impact, assessing upper extremity function is important for monitoring disease severity [6]. Although upper extremity function strongly impacts QoL and is a critical measurement for patients with pronounced disability, it only recently started gaining importance in therapeutic trials [7].

Different clinical assessments are currently available to measure upper extremity function, or manual dexterity, including the strength–dexterity test [8], the Grooved Pegboard [9], the Minnesota Dexterity Test (and its turning subtest) [10], the Functional Dexterity Test [11,12], and the Nine-Hole Peg Test (9HPT) [6]. The 9HPT is commonly used for assessing upper extremity function due to its convenience and favorable psychometric properties, and it is included in the Multiple Sclerosis Functional Composite [6,13,14]. However, functional assessments (eg, the 9HPT) require additional equipment and time in the clinic and thus are infrequently administered, thereby limiting their utility [15]. New assessments are needed, allowing minimally burdensome remote assessment and more frequent administration.

In response to that need, neurology research started shifting to digital health technologies—tools such as various types of sensors and wearables that could allow real-time monitoring of disease symptoms [16]. In an analysis of trials investigating epilepsy, MS, Alzheimer disease, and Parkinson disease on ClinicalTrials.gov, it was revealed that different “mobile applications” were referenced in 35.1% of the investigations, with “smartphone” being the second most frequently referenced digital health technology (17.2%) after “wearables” (29.3%) [16]. Therefore, given its convenient functionality and omnipresence (86.3% of the global population owns a smartphone) [17], a smartphone appears to be an optimal platform for digital MS assessments [16,18].

The Pinching Test was designed as an objective, ecologically valid (ie, reflective of typical everyday life) [19], smartphone sensor–based assessment of upper extremity function that could be performed in a clinic or independently at home [20]. Unlike the 9HPT, which uses a single summary time–based score, the Pinching Test measures multiple characteristics of upper extremity movement using smartphone-based sensors and was first deployed in the clinical trial “Monitoring of Multiple Sclerosis Participants With the Use of Digital Technology (Smartphones and Smartwatches)—A Feasibility Study” (ClinicalTrials.gov NCT02952911) [21]. The number of successful pinches and double touch asynchrony, 2 of the features derived from the Pinching Test, showed moderate-to-good test-retest reliability and correlated with the 9HPT; Expanded Disability Status Scale (EDSS) [22]; arm-related items of the 29-item Multiple Sclerosis Impact Scale (MSIS-29) [23]; and whole brain volume [20].

Going beyond the initial pinching movement analysis, the sensor data from the Pinching Test include a rich array of features potentially providing information on the quality of limb movement. Thus, we investigated additional Pinching Test features specifically assessing muscle weakness, spasticity, and tremor potentially related to motor and sensory deficits [24] and provide greater insights into the characteristics of the pinching motion (eg, pinching smoothness, pinching precisions, etc). Here, we assess this expanded feature space’s test-retest reliability, its agreement with the standard clinical measures of MS disease state, and its ability to differentiate people with MS from healthy controls (HCs). We also evaluate the shared and complementary interfeature information.

## Methods

### Study Design

This 24-week, prospective study (ClinicalTrials.gov NCT02952911) assessed the feasibility of remotely monitoring MS with the Floodlight Proof-of-Concept (PoC) app on a provisioned smartphone (Samsung Galaxy S7). The full study design and inclusion and exclusion criteria were previously reported [21]. In total, 76 people with MS and 25 HCs aged 18-55 years were enrolled across 2 sites (Multiple Sclerosis Centre of Catalonia, Vall d’Hebron University Hospital, Barcelona, Spain; University of California San Francisco, San Francisco, California, United States). People with MS were diagnosed with the 2010 revised McDonald criteria [25] (treated or untreated) and had a baseline EDSS score between 0.0 and 5.5.

### Clinical Assessments

Participants attended 3 clinic visits (baseline, week 12, and week 24 [end of study]) where they underwent standard clinical measures of disability. People with MS were evaluated by a range of commonly used measures, including the 9HPT, EDSS, MSIS-29 symptom questionnaire, oral Symbol Digit Modalities Test (SDMT), and Fatigue Scale for Motor and Cognitive Functions (FSMC), while HCs were evaluated with the 9HPT, oral SDMT, and FSMC. In addition, during the baseline visit, all study participants were instructed on performing daily smartphone-based tests, including the Pinching Test, on the provisioned smartphone with the Floodlight PoC app preinstalled.

### Pinching Test

**Figure 1:**
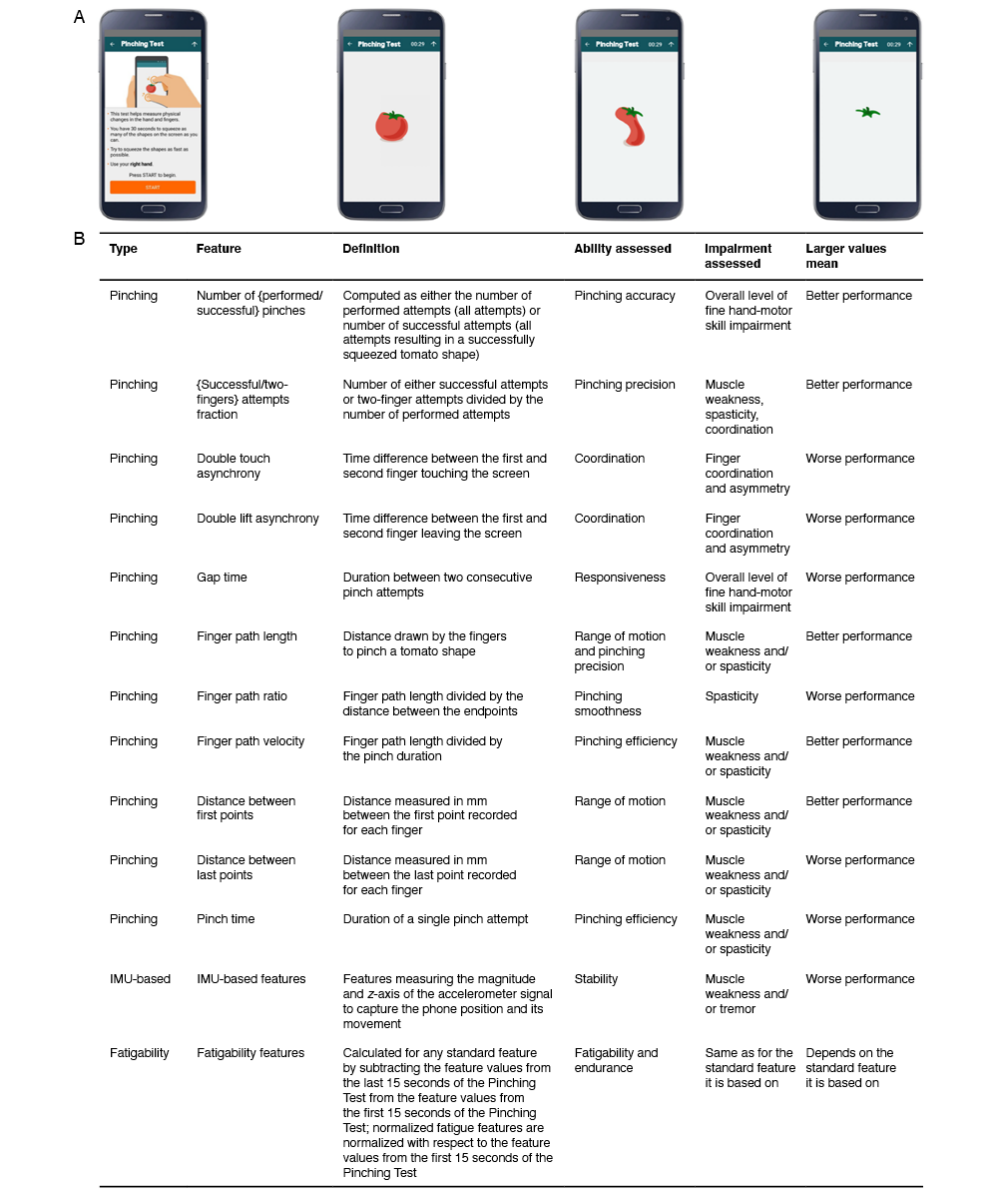
The Pinching Test. (A) Images of what the smartphone screen displays during the Pinching Test. Participants were instructed to squeeze, or pinch, as many tomato shapes as possible within 30 seconds with 2 fingers while holding the smartphone in the stabilizing hand. The dominant and nondominant hands were tested in consecutive tests. (B) Details of the pinching, IMU-based, and fatigability features comprising the Pinching Test that were extracted to assess the hand-motor abilities of people with MS, including pinching accuracy, pinching precision and range of motion, coordination, responsiveness, pinching smoothness, pinching efficiency, stability, and endurance. IMU: inertial measurement unit; MS: multiple sclerosis.

The Pinching Test examines upper extremity function [20]. By evaluating the coordination of 2 fingers (the thumb and either the second or third finger), it relates to the patient’s ability to grasp small objects (eg, keys, pens, and door handles). Participants performing the test hold their smartphones in 1 hand and use the other hand to pinch or squeeze as many tomato shapes on the screen as possible in 30 seconds (Figure 1A). It thus likely involves motor, cerebellar, visual, and cognitive aspects of upper extremity function. After successfully pinching the first tomato shape using 2 fingers of the tested hand, a new tomato shape appears on the smartphone display at a random location. The dominant and nondominant hands were assessed in alternate test runs.

### Feature Extraction

In total, 13 pinching features, 11 inertial measurement unit (IMU)–based features (ie, smartphone acceleration and orientation), and 13 fatigability features that are illustrative of the test were extracted from the raw touchscreen and accelerometer signals (the full mapping of the interfeature relations, as well as feature descriptions, can be seen in Figure 1B. For feature definitions, see Table S1 in Multimedia Appendix 1).

Pinching features capture the overall upper extremity impairment (number of performed pinches, number of successful pinches, fraction of successful attempts, and fraction of two-finger attempts); finger coordination (double touch asynchrony and double lift asynchrony); responsiveness (gap time); range of motion or precision (finger path length); as well as muscle weakness, spasticity, or tremor (finger path ratio, finger velocity, distance between first or last points, pinch time). Pinching features were preprocessed by resampling the signal at a sampling frequency (fs) of 60 Hz to uniformize its sampling rate. The resampled signal was then filtered via the Butterworth fourth order low-pass filter at a cut-off frequency of fs/2.

IMU-based features are based on either the mean, SD, or kurtosis of the accelerometer magnitude of the untested (ie, stabilizing) hand holding the smartphone device or the smartphone’s orientation and aim to capture coordination signals between the 2 hands, muscle weakness, or tremor. IMU-based features were preprocessed by removing 2 seconds at the beginning and at the end of each text execution (to ensure that data come only from test execution attempts rather than the set-up stage where participants might still be positioning themselves). The collected data were then resampled to the frequency of 20 milliseconds, with a moving Blackman window of 10 samples applied to smooth the data.

Fatigability features are computed for each pinching feature by calculating the performance difference between the second and first half of the test. The test data were split equally to achieve comparable variability. As the fatigability features are based on the pinching features, they have not been additionally preprocessed.

### Data Processing

As the Pinching Test is unsupervised, individual test runs not performed in accordance with the test’s instructions needed to be identified [20]. Study participants were instructed to hold the phone in the stabilizing hand while taking the Pinching Test; test runs characterized by the phone lying on a hard surface, such as a table, were considered invalid [20]. Furthermore, to enable a meaningful assessment of upper extremity function, only study participants who contributed ≥20 valid test runs were retained for the analyses.

For the test-retest reliability analyses, Pinching Test features were aggregated by computing the median feature value across 2-week windows (a 2-week period is long enough to minimize potential variability due to nondisease-related factors, eg, weekdays vs weekends, patient’s good vs bad days [20], and short enough for MS to remain stable). At least 3 valid individual assessments were required for each 2-week window. The 2-week time frame was chosen to decrease general disease-independent variability attributable to differences between weekdays and weekends or changes in the patient’s well-being. Since fatigue levels in people with MS can fluctuate daily [26], fatigability features were additionally aggregated by taking the SD across the 2-week windows.

Similarly, for all other cross-sectional analyses, Pinching Test features were aggregated by either their median (all features) or SD (fatigability features), but the data were aggregated across the whole study duration. Additionally, standard clinical measures such as 9HPT, EDSS, oral SDMT, MSIS-29 arm (items 2, 6, and 15), FSMC (total score, physical subscale, and cognitive subscale) were aggregated by taking the mean across the 3 clinic visits at baseline, week 12, and week 24 (end of study).

### Statistical Analysis

Four main statistical analyses were conducted, including (1) test-retest reliability; (2) age- and sex-adjusted Spearman rank correlation (adjusted using a linear model); (3) age- and sex-adjusted known-groups validity; and (4) a repeated-measures correlation [27], principal component, and factor analyses. Although the data from the dominant and nondominant hands were alike (Figure S1 in Multimedia Appendix 1), the variability was slightly lower for the dominant hand data; therefore, only the analyses conducted on the dominant hand are reported. Further supporting this choice is the lack of a difference in the ability between the dominant and nondominant hands to differentiate between people with MS with no-to-minimal disability and people with MS with at least mild disability on either the pyramidal functional system (pyramidal functional system score [FSS] ≤2 vs ≥3) or on the cerebellar functional system (cerebellar FSS ≤1 vs ≥2) (Tables S2 and S3 in Multimedia Appendix 1).

The test-retest reliability was assessed by computing intraclass correlation coefficients [second model, first type, ICC(2,1)] including all consecutive 2-week windows [20]. A minimum of 3 valid test runs were required for each 2-week window, and test-retest reliability was considered as poor [ICC(2,1)<0.5], moderate [ICC(2,1)=0.5 to 0.74], good [ICC(2,1)=0.75-0.9], or excellent [ICC(2,1)>0.9] [28].

The age- and sex-adjusted nonparametric Spearman rank correlation analysis evaluated the agreement with the 9HPT, EDSS, pyramidal and cerebellar FSS, MSIS-29 arm items, oral SDMT (multiplied by –1, thus higher scores equal worse performance on all clinical anchors), and FSMC. This analysis was limited to people with MS only as both EDSS and MSIS-29 were not collected in HCs. The correlation strength was considered as uncorrelated (|*r*|<0.25), fair (|*r*|=0.25 to 0.49), moderate-to-good (|*r*|=0.50 to 0.75), or good-to-excellent (|*r*|>0.75) [29]. In addition, 2 separate age- and sex-adjusted partial Spearman rank correlation analyses were conducted on: (1) the pinching features, 9HPT, and oral SDMT to assess our hypothesis that the pinching features are primarily driven by a motor component rather than a cognitive or overall MS disease severity component affecting both the 9HPT and oral SDMT, and (2) the fatigability features, 9HPT, and FSMC to study whether the fatigability features primarily measure upper extremity function or total fatigue (including motor and cognitive fatigue) or both.

The age- and sex-adjusted known-groups validity analysis assessed the ability to differentiate between HCs and people with MS subgroups and was evaluated using the Mann-Whitney *U* test (after false discovery rate correction was applied to each feature category: pinching, IMU-based, fatigability aggregated by median, fatigability aggregated by SD), Cohen *d* effect size, and the area under the receiver operating curve (AUC). Two subgroups of people with MS were included: people with MS with normal 9HPT time at baseline (people with MS-Normal) and people with MS with abnormal 9HPT time at baseline (people with MS-Abnormal). The threshold for abnormal 9HPT time was mean+2 SDs of the dominant-handed normative data of HCs [30]. Thus, all people with MS with a baseline 9HPT time below 22.15 seconds for the dominant hand were considered as people with MS-Normal, while all remaining people with MS were considered as people with MS-Abnormal (the 9HPT was chosen as the reference as we aimed to differentiate people with MS with aberrant upper extremity impairment, ie, people with MS-Abnormal, from people with MS with normal upper extremity function, ie, people with MS-Normal, and HCs). In a separate analysis, the ability of the fatigability features to differentiate between people with MS with and without fatigue, defined as at least mild fatigue (≥43 points on the FSMC) [31], was studied.

To evaluate the shared and complementary information between the Pinching Test features, a repeated-measures correlation analysis was performed, estimating an independent intercept for each subject, thereby minimizing potential bias introduced by differences in disease severity between subjects [27]. This was complemented by principal component and factor analyses.

Statistical significance was set at *P*<.05. Analyses were performed in Python (including the following packages: NumPy, SciPy, PyMC3 [for ICC values], and pingouin [for repeated-measures correlations and partial correlations]) [32-35].

### Ethical Considerations

All participants provided written informed consent, and ethical approval was obtained from the ethics committee of the Multiple Sclerosis Centre of Catalonia, Vall d’Hebron University Hospital, Barcelona, Spain [study: PR(AG)300/2016] and the institutional review board of the University of California San Francisco, San Francisco, California, United States (reference: 175728) prior to study initiation. The study was registered on ClinicalTrials.gov (NCT02952911).

## Results

### Study Population and Adherence

We enrolled 76 people with MS and 25 HCs in the study, of which, 67 people with MS and 18 HCs met inclusion criteria for the analyses. The baseline demographics and participants’ disease characteristics (Table 1) resembled those of the full study population [21]. The assessed people with MS had mostly mild disease with limited upper extremity functional impairment, with a mean EDSS score of 2.4 (SD 1.4) and a mean 9HPT time of 22.4 (SD 4.2) seconds for both hands and 22.3 (SD 4.7) for the dominant hand. Compared with HCs, the people with MS cohort included more female participants (67% vs 33%) and had a higher mean age (39.3, SD 7.8 years vs 35, SD 8.9 years). Data on participants’ previous disease-modifying treatment have already been published [21]. EDSS and 9HPT time remained stable during the study; changes from baseline to week 24 were mostly within 1 point on the EDSS (Figures S2A and B in Multimedia Appendix 1) and mostly within 20% on the 9HPT (Figures S2C and D in Multimedia Appendix 1).

**Table 1:** Baseline demographics and disease characteristics^a^.

Variable		HCs^b^ (n=18)	People with MS^c^ (n=67)
Age (years), mean (SD)		35.0 (8.9)	39.3 (7.8)
Female, n (%)		6 (33)	45 (67)
**Diagnosis, n (%)**			
	Relapsing-remitting multiple sclerosis	N/A^d^	60 (90)
	Primary progressive multiple sclerosis	N/A	3 (4)
	Secondary progressive multiple sclerosis	N/A	4 (6)
Time since diagnosis (years), mean (SD)		N/A	9.1 (6.4)
EDSS^e^ score, mean (SD)		N/A	2.4 (1.4)
9HPT^f^ time (both hands) (seconds), mean (SD)		18.8 (1.7)	22.4 (4.2)
9HPT time (dominant hand) (seconds), mean (SD)		18.7 (2.0)	22.3 (4.7)
9HPT time (nondominant hand) (seconds), mean (SD)		19.0 (1.8)	22.5 (4.4)
SDMT^g^, number of correct responses, mean (SD)		64.6 (8.3)	53.9 (12.1)
MSIS-29^h^ arm-related items, mean (SD)^i^		N/A	24.7 (26.1)
FSMC^j^ total score, mean (SD)		25.6 (6.3)	58.8 (23.0)
FSMC physical subscale score, mean (SD)		12.6 (3.0)	30.7 (11.7)
FSMC cognitive subscale score, mean (SD)		13.1 (3.6)	28.6 (11.9)

^a^The full baseline demographics and disease characteristics have been previously reported [21].

^b^HCs: healthy controls.

^c^MS: multiple sclerosis.

^d^N/A: not available.

^e^EDSS: Expanded Disability Status Scale.

^f^9HPT: Nine-Hole Peg Test.

^g^SDMT: Symbol Digit Modalities Test.

^h^MSIS-29: 29-item Multiple Sclerosis Impact Scale.

^i^Items 2, 6, and 15.

^j^FSMC: Fatigue Scale for Motor and Cognitive Functions.

### Test-Retest Reliability

**Figure 2:**
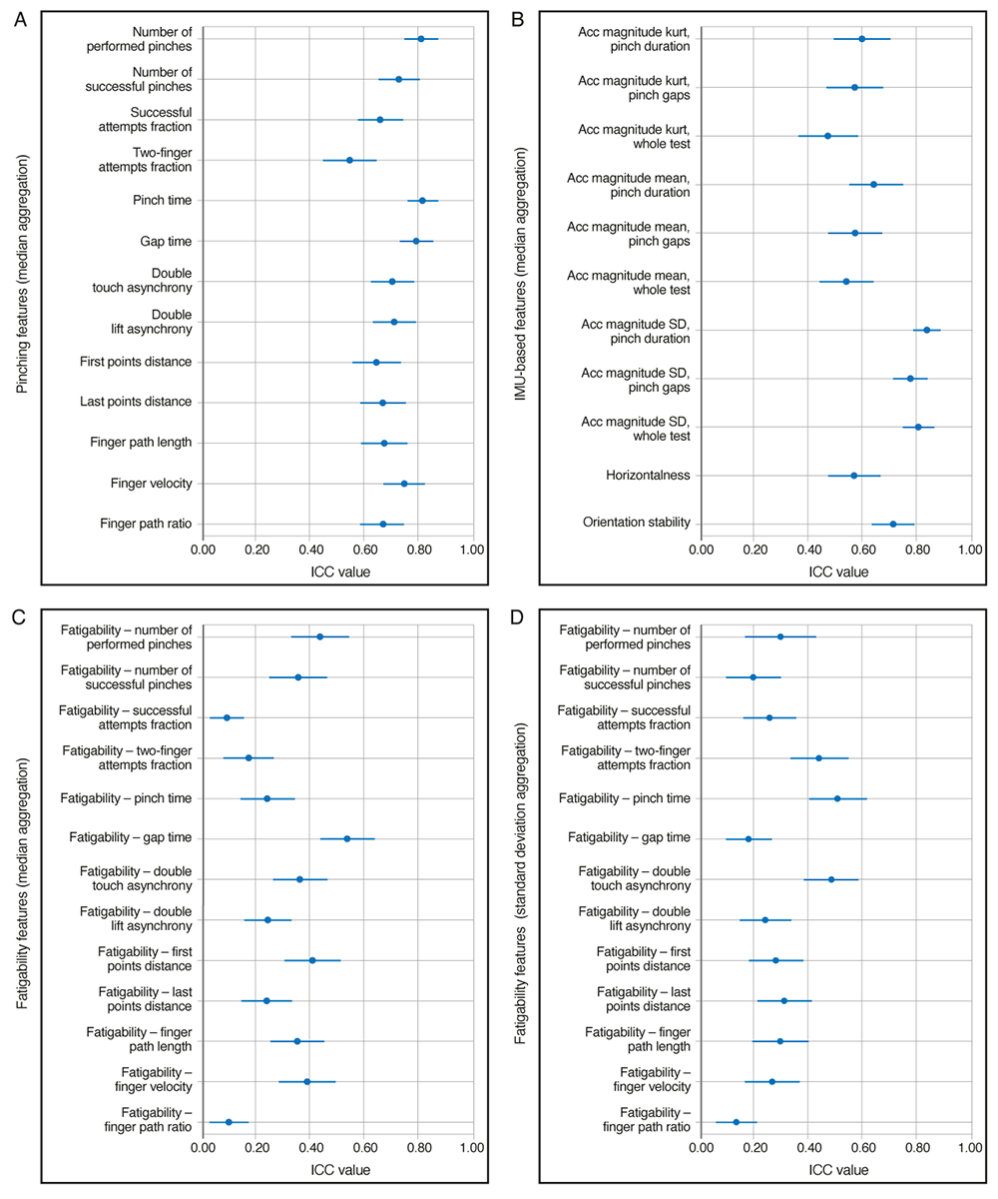
Test–retest reliability in people with MS. ICC(2,1) values of (A) pinching, (B) IMU-based, and (C,D) fatigability features. All consecutive 2-week windows with at least 3 valid test runs (per study participant) were included in the analyses. Feature values were aggregated across the 2-week windows by taking the (A-C) median or (D) SD. Error bars indicate the 95% CI estimated by bootstrapping. Acc: accelerometer; ICC: intraclass correlation coefficient; IMU: inertial measurement unit; kurt: kurtosis; MS: multiple sclerosis.

For test-retest reliability analysis for people with MS (Figure 2), pinching features showed moderate or good test-retest reliability, with ICC(2,1) between 0.54 and 0.81. The ICC(2,1) for the 9HPT time on the dominant hand across the 3 clinic visits was 0.83. IMU-based features, reflecting data collected with the stabilizing hand, showed similar ICC(2,1), ranging within 0.47-0.84. In contrast, fatigability features were mostly unreliable [ICC(2,1)≤0.5], irrespective of the aggregation method, as expected given the short test duration. Only fatigability gap time computed with the median aggregation method [ICC(2,1)=0.54] and fatigability pinch time computed with the SD aggregation method [ICC(2,1)=0.51] showed moderate test-retest reliability. Across all features, ICC(2,1) values were generally smaller in HCs, possibly because of lower intersubject variability in this cohort (Figure S3 in Multimedia Appendix 1).

### Correlation Analyses

**Figure 3:**
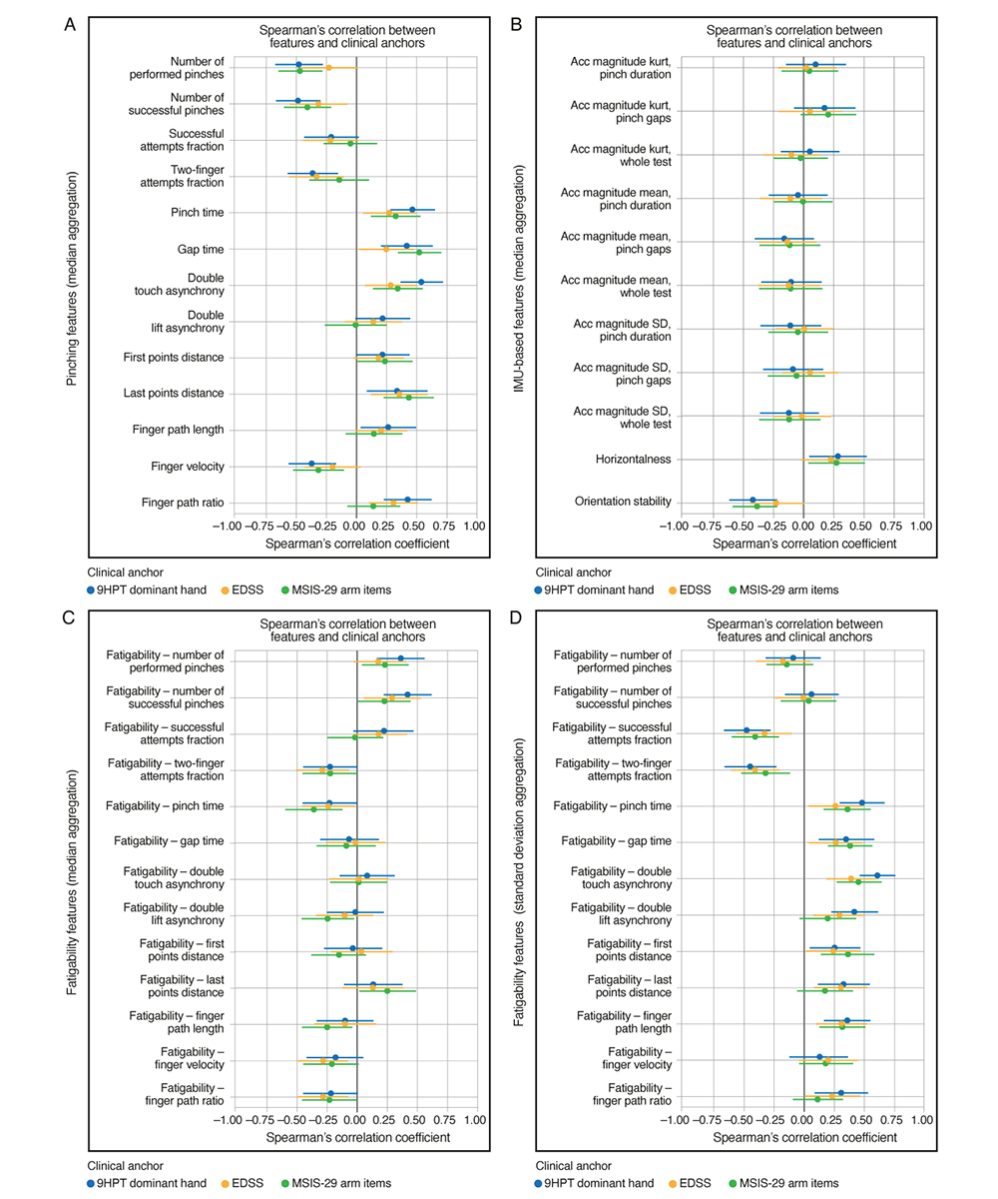
Cross-sectional Spearman rank correlations between Pinching Test features and standard clinical measures of upper extremity function and overall disease severity in people with MS. (A) Pinching, (B) IMU-based, and (C,D) fatigability features were correlated against dominant-handed 9HPT time (blue), EDSS score (orange), and MSIS-29 arm items (green) after adjusting for age and sex. Error bars indicate the 95% CI estimated by bootstrapping. 9HPT: Nine-Hole Peg Test; Acc: accelerometer; EDSS: Expanded Disability Status Scale; IMU: inertial measurement unit; kurt: kurtosis; MS: multiple sclerosis; MSIS-29 arm: 29-item Multiple Sclerosis Impact Scale items 2, 6, and 15.

**Figure 4:**
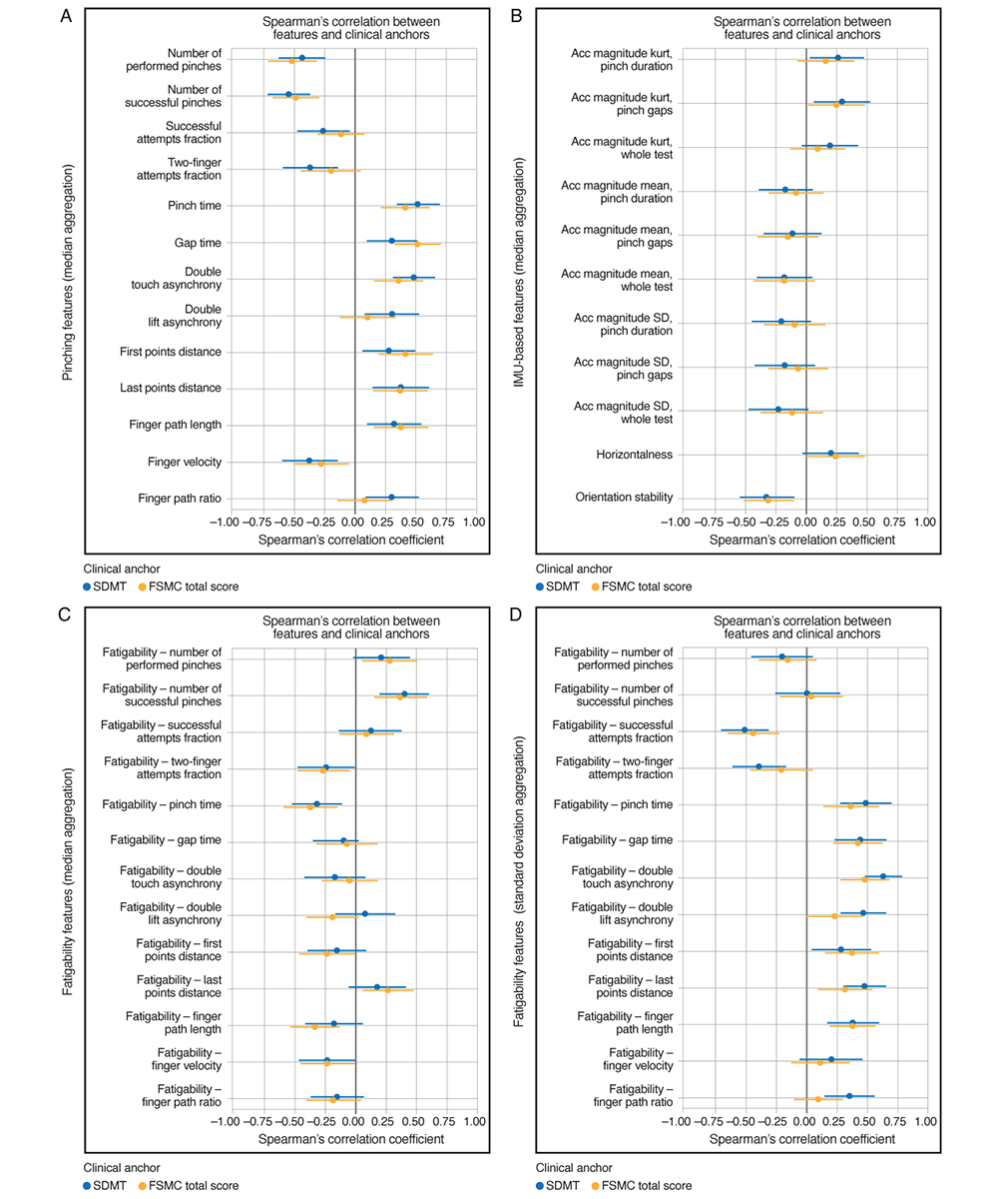
Cross-sectional Spearman rank correlations between Pinching Test features and standard clinical measures of information processing speed and fatigue in people with MS. (A) Pinching, (B) IMU-based, and (C,D) fatigability features were correlated against the number of correct responses on the oral SDMT (blue) and FSMC total score (orange) after adjusting for age and sex. The number of correct responses on the oral SDMT were multiplied by −1 so that higher scores on both the oral SDMT and FSMC indicate worse performance. Error bars indicate the 95% CI estimated by bootstrapping. Acc: accelerometer; FSMC: Fatigue Scale for Motor and Cognitive Functions; IMU: inertial measurement unit; kurt: kurtosis; MS: multiple sclerosis; SDMT: Symbol Digit Modalities Test.

Most pinching features showed either fair or moderate-to-good correlations with the standard clinical measures of upper extremity function and overall disease severity (Figures 3 and S4). Strongest agreement with the 9HPT was observed for double touch asynchrony (*r*=0.54), number of successful pinches (*r*=–0.48), and number of performed pinches (*r*=–0.47). Seven additional pinching features—two-finger attempts fraction, pinch time, gap time, last points distance, finger path length, finger velocity, and finger path ratio—showed fair correlation with the 9HPT (|*r*|=0.26-0.47). However, 3 pinching features—double lift asynchrony, first points distance, and successful attempts fraction—did not correlate with the 9HPT (all |*r*|=0.21-0.22). Regarding other standard clinical measures, at least half of the pinching features showed fair correlations with the EDSS (|*r*|=0.25-0.36 for 7/13 features), cerebellar FSS (|*r*|=0.25-0.46 for 9/13 features), and fair or moderate-to-good correlations with MSIS-29 arm items (|*r*|=0.31-0.52 for 7/13 features). Associations with pyramidal FSS were generally weaker, with only 3 out of 13 features showing fair correlations (|*r*|=0.28-0.30). Pinching features were also associated with information processing speed and fatigue (Figure 4); all 13 pinching features showed fair or moderate-to-good correlations with the oral SDMT (|*r*|=0.26-0.55), and most pinching features (9/13) correlated with FSMC total score, reaching fair or moderate-to-good strength (|*r*|=0.28-0.52).

In comparison, only 1 IMU-based feature—orientation stability—consistently showed fair correlations with clinical measures (9HPT: *r*=–0.41; MSIS-29 arm items: *r*=–0.38; oral SDMT: *r*=–0.33; FSMC total score: *r*=–0.31) (Figures 3 and 4).

Fatigability features were generally associated with clinical measures of upper extremity function and overall disease severity, particularly when applying the SD aggregation (Figures 3 and S4). Using this aggregation method, correlations with 9HPT time (|*r*|=0.26-0.61 for 10/13 features) and cerebellar FSS (|*r*|=0.26-0.52 for 8/13 features) reached fair or moderate-to-good strength for most fatigability features, while correlations with EDSS (|*r*|=0.26-0.41 for 8/13 features), pyramidal FSS score (|*r*|=0.25-0.37 for 7/13 features), and MSIS-29 arm items (|*r*|=0.32-0.46 for 7/13 features) were fair. Fatigability features aggregated by taking the SD were also associated with information processing and fatigue (Figure 4). Most fatigability features showed fair or moderate-to-good correlations with the oral SDMT (|*r*|=0.28-0.62 for 10/13 features), while correlations with the FSMC total score reached fair strength (|*r*|=0.31-0.47 for 7/13 features).

Across pinching, IMU-based, and fatigability features, correlations with FSMC physical and cognitive subscales resembled those with FSMC total score (Figure S5 in Multimedia Appendix 1).

**Figure 5:**
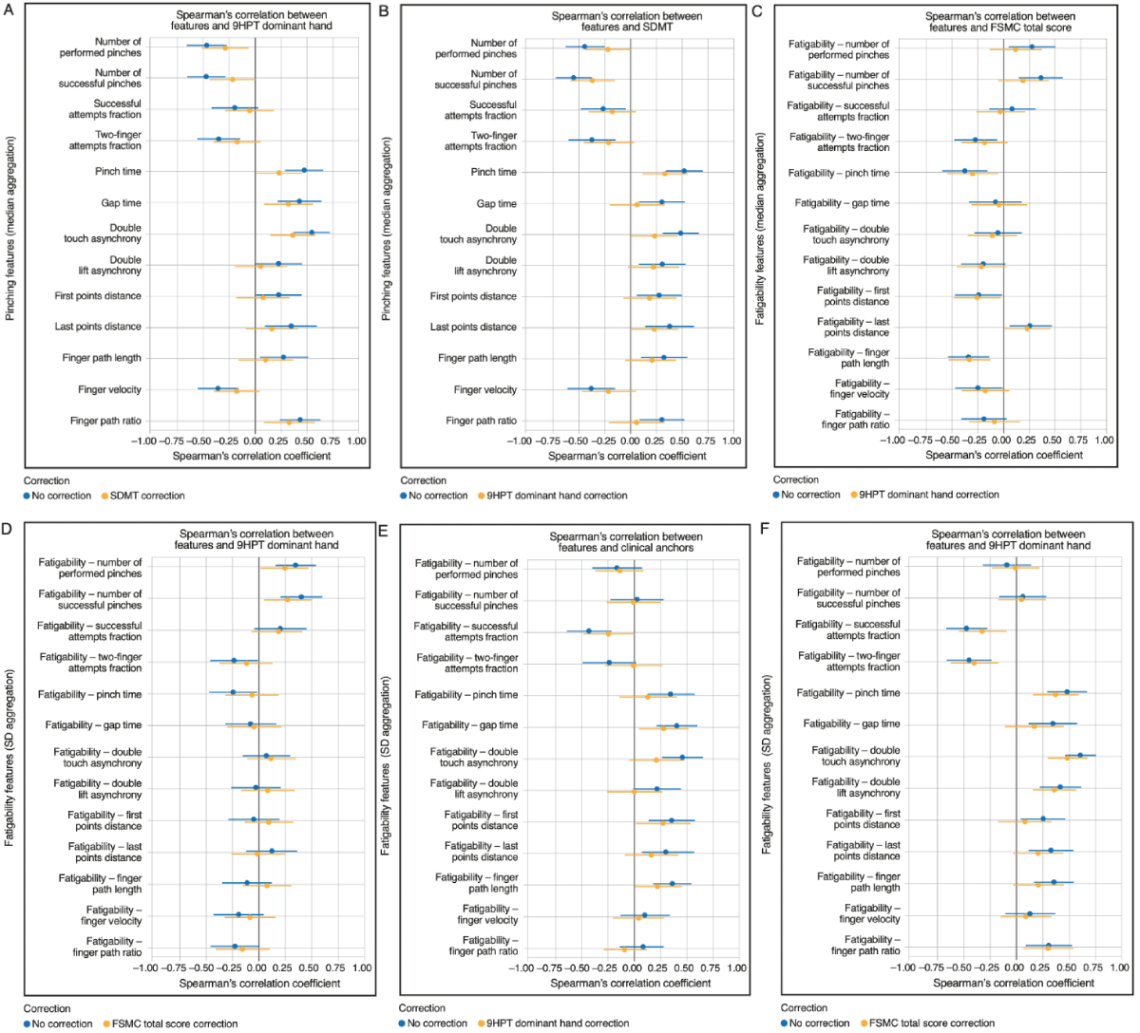
Partial Spearman rank correlations between Pinching Test features and standard clinical measures in people with MS. (A,B) Partial correlation between pinching features and dominant-handed 9HPT time (A) and number of correct responses on the oral SDMT (B) after adjusting for age and sex. Number of correct responses on the oral SDMT were multiplied with –1 so that higher scores on both the oral SDMT and 9HPT indicate worse performance. Four pinching features—number of performed pinches (*r*=–0.29), double touch asynchrony (*r*=0.36), gap time (*r*=0.31), and finger path ratio (*r*=0.32)—retained a significant correlation with 9HPT time even after adjusting for the oral SDMT (A), but their correlations with the oral SDMT tended to zero after accounting for dominant-handed 9HPT time (B). This suggests that these features are primarily driven by a motor component. (C-F) Partial correlation analysis between fatigability features and FSMC total score (C,E) and dominant-handed 9HPT time (D,F), for median aggregation (C,D) and SD aggregation method (E,F). (C) With the median aggregation method, only fatigability pinch time (*r*=–0.30), fatigability path length (*r*=–0.33), and fatigability first points distance (*r*=–0.25) retained their correlations with FSMC total score after accounting for dominant-handed 9HPT. (D) When applying the SD aggregation method instead, only fatigability gap time (*r*=0.29) and fatigability points distance (*r*=0.28) correlated with FSMC total score after accounting for dominant-handed 9HPT time. Orange bars indicate the correlation after adjustment; blue bars indicate the correlation without adjustment. Error bars indicate the 95% CI estimated by bootstrapping. 9HPT: Nine-Hole Peg Test; FSMC: Fatigue Scale for Motor and Cognitive Functions; MS: multiple sclerosis; SDMT: Symbol Digit Modalities Test.

The subsequent partial correlation analysis revealed the primary drivers of the pinching features (motor vs cognitive), showing that these features primarily capture motor impairment over cognitive impairment (Figures 5A and B). Specifically, the number of performed pinches (*r*=–0.29), double touch asynchrony (*r*=0.36), gap time (*r*=0.31), and finger path ratio (*r*=0.32) all correlated with 9HPT time after accounting for the number of correct responses on the oral SDMT, but their correlations with the oral SDMT tended toward zero after accounting for 9HPT time (*r*=–0.22, *r*=0.23, *r*=0.06, and *r*=0.06, respectively). A separate partial correlation analysis assessed whether the fatigability features primarily capture fatigue or upper extremity function (Figures 5C-F). Noticeably, when using the median aggregation method, fatigability pinch time (*r*=–0.30), fatigability path length (*r*=–0.33), and fatigability first points distance (*r*=–0.25) correlated with the FSMC total score even after accounting for 9HPT time. Similarly, 2 fatigability features correlated with FSMC total score after accounting for 9HPT time when applying the SD aggregation method instead (fatigability gap time: *r*=0.29; fatigability first points distance: *r*=0.28). Contrastingly, these features did not correlate with 9HPT time after accounting for FSMC total score.

### Ability to Differentiate and Distinguish Between HCs and People With MS Subgroups

The ability of the Pinching Test features to differentiate among HCs, people with MS-Normal, and people with MS-Abnormal varied depending on the feature type (Tables 2-5). Overall, pinching features best differentiated between people with MS-Normal and people with MS-Abnormal (Table 2). For the 8 pinching features that showed a statistically significant difference between the 2 subgroups (table cells with italic formatting; *P*<.05), AUC ranged from 0.68 to 0.78 and Cohen *d* from 0.43 to 1.04. Additionally, 3 of these features differentiated between HCs and people with MS-Abnormal (AUC=0.75-0.75; Cohen *d*=0.35-0.79; *P*<.05 for all 3 features).

In contrast, none of the IMU-based features (Table 3) and none of the fatigability features (Table 4) when using the median aggregation method differentiated between the groups (all *P*>.05). However, a few fatigability features differentiated between people with MS-Normal and people with MS-Abnormal after aggregating by SD (Table 5). In the 5 fatigability features that showed a statistically significant difference between these 2 subgroups (table cells with italic formatting; *P*<.05), AUC ranged from 0.70 to 0.82 and Cohen *d* from 0.37 to 1.10 (Tables 2-5). Three of these features also differentiated between people with MS-Abnormal and HCs (AUC=0.74-0.76; Cohen *d*=0.52-0.64; *P*<.05 for all 3 features). Two fatigability features (fatigability gap time and fatigability double touch asynchrony) differentiated between people with MS without and people with MS with at least mild levels of fatigue on the FSMC when using the SD aggregation method (AUC=0.72 and 0.73; Cohen *d*=0.63 and 0.50, respectively; *P*<.05 for both) (Tables S4 and S5 in Multimedia Appendix 1).

**Table 2:** Ability of the pinching features (aggregated by median) to differentiate and distinguish between HCs^a^ and people with MS^b^ subgroups^c^.

Feature	HCs vs people with MS-Normal			HCs vs people with MS-Abnormal			People with MS-Normal vs people with MS-Abnormal		
	*P* value^d^	AUC^e^	Cohen *d*	*P* value^d^	AUC	Cohen *d*	*P* value^d^	AUC	Cohen *d*
Number of performed pinches	.76	0.53	0.09	.14	0.67	0.72	*.02* ^f^	*0.70*	*0.76*
Number of successful pinches	.81	0.52	0.22	.06	0.71	0.66	*.01*	*0.75*	*0.98*
Successful attempts fraction	.61	0.56	0.28	.47	0.59	0.35	.08	0.66	0.67
Two-finger attempts fraction	.41	0.60	0.35	*.02*	*0.75*	*0.35*	*.02*	*0.70*	*0.91*
Pinch time	.61	0.56	0.07	.47	0.58	0.36	*.04*	*0.68*	*0.43*
Gap time	.47	0.58	0.35	*.02*	*0.75*	*0.79*	*.03*	*0.69*	*0.71*
Double touch asynchrony	.74	0.54	0.22	*.02*	*0.75*	*0.78*	*<.001*	*0.78*	*1.04*
Double lift asynchrony	.87	0.52	0.33	.47	0.58	0.08	.41	0.59	0.39
First points distance	.75	0.54	0.06	.62	0.56	0.28	.41	0.59	0.33
Last points distance	.26	0.63	0.53	.47	0.59	0.32	*.02*	*0.73*	*0.86*
Finger path length	.47	0.58	0.42	.74	0.54	0.25	.23	0.62	0.37
Finger velocity	≥.99	0.50	0.02	.47	0.59	0.41	.34	0.60	0.40
Finger path ratio	.47	0.58	0.44	.41	0.61	0.29	*.02*	*0.72*	*0.93*

^a^HC: healthy control.

^b^MS: multiple sclerosis.

^c^This analysis included 18 HCs, 38 people with MS–Normal (ie, with a baseline Nine-Hole Peg Test time below 22.15 seconds), and 29 people with MS-Abnormal (ie, with a baseline Nine-Hole Peg Test time above 22.15 seconds).

^d^Mann-Whitney *U* test with false discovery rate correction adjusted for age and sex.

^e^AUC: area under the curve.

^f^Italic formatting represents a statistically significant *P* value of <.05.

**Table 3:** Ability of the IMU^a^-based features (aggregated by median) to differentiate and distinguish between HCs^b^ and people with MS^c^ subgroups^d^.

Feature	HCs vs people with MS-Normal			HCs vs people with MS-Abnormal			People with MS-Normal vs people with MS-Abnormal		
	*P* value^e^	AUC^f^	Cohen *d*	*P* value^e^	AUC	Cohen *d*	*P* value^e^	AUC	Cohen *d*
Acc^g^ magnitude kurt^h^, pinch duration	.95	0.53	0.25	≥.99	0.51	0.38	≥.99	0.50	0.15
Acc magnitude kurt, pinch gaps	.95	0.54	0.25	.57	0.62	0.40	.34	0.63	0.02
Acc magnitude kurt, whole test	.95	0.56	0.27	.95	0.58	0.38	.95	0.53	0.14
Acc magnitude mean, pinch duration	.46	0.63	0.49	.95	0.56	0.34	.95	0.56	0.22
Acc magnitude mean, pinch gaps	.95	0.54	0.09	.95	0.57	0.33	.29	0.65	0.45
Acc magnitude mean, whole test	.68	0.61	0.45	≥.99	0.52	0.02	.30	0.64	0.50
Acc magnitude SD, pinch duration	≥.99	0.51	0.01	.95	0.55	0.03	.95	0.52	0.02
Acc magnitude SD, pinch gaps	.95	0.52	0.04	.95	0.57	0.18	.95	0.54	0.13
Acc magnitude SD, whole test	≥.99	0.50	0.01	.95	0.53	0.08	.95	0.53	0.09
Horizontalness	.95	0.53	0.16	.34	0.66	0.62	.25	0.67	0.67
Orientation stability	≥.99	0.50	0.02	.29	0.69	0.58	.19	0.70	0.63

^a^IMU: inertial measurement unit.

^b^HC: healthy control.

^c^MS: multiple sclerosis.

^d^This analysis included 18 HCs, 38 people with MS–Normal (ie, with a baseline Nine-Hole Peg Test time below 22.15 seconds), and 29 people with MS-Abnormal (ie, with a baseline Nine-Hole Peg Test time above 22.15 seconds).

^e^Mann-Whitney *U* test with false discovery rate correction adjusted for age and sex.

^f^AUC: area under the curve.

^g^Acc: accelerometer.

^h^Kurt: kurtosis.

**Table 4:** Ability of the fatigability features (aggregated by median) to differentiate and distinguish between HCs^a^ and people with MS^b^ subgroups^c^.

Feature	HCs vs people with MS-Normal			HCs vs people with MS-Abnormal			People with MS-Normal vs people with MS-Abnormal		
	*P* value^d^	AUC^e^	Cohen *d*	*P* value^d^	AUC	Cohen *d*	*P* value^d^	AUC	Cohen *d*
Fatigability number of performed pinches	.54	0.59	0.24	.64	0.58	0.37	.17	0.67	0.61
Fatigability number of successful pinches	.84	0.53	0.09	.49	0.63	0.55	.16	0.69	0.69
Fatigability successful attempts fraction	.53	0.61	0.27	.62	0.58	0.26	.85	0.52	0.04
Fatigability two-finger attempts fraction	.53	0.61	0.42	.17	0.70	0.08	.26	0.64	0.17
Fatigability pinch time	.84	0.53	0.31	.17	0.71	0.01	.16	0.69	0.49
Fatigability gap time	.53	0.61	0.38	.84	0.53	0.11	.84	0.54	0.31
Fatigability double touch asynchrony	.53	0.61	0.36	.53	0.60	0.05	.91	0.51	0.15
Fatigability double lift asynchrony	.77	0.55	0.42	.91	0.51	0.43	.87	0.52	0.13
Fatigability first points distance	.85	0.53	0.07	.53	0.60	0.34	.72	0.56	0.23
Fatigability last points distance	.81	0.55	0.05	.89	0.52	0.17	.72	0.56	0.22
Fatigability finger path length	.84	0.54	0.07	.37	0.66	0.23	.49	0.61	0.38
Fatigability finger velocity	.77	0.56	0.10	.54	0.60	0.28	.49	0.61	0.36
Fatigability finger path ratio	.84	0.54	0.43	.53	0.61	0.06	.17	0.66	0.52

^a^HC: healthy control.

^b^MS: multiple sclerosis.

^c^This analysis included 18 HCs, 38 people with MS–Normal (ie, with a baseline Nine-Hole Peg Test time below 22.15 seconds), and 29 people with MS-Abnormal (ie, with a baseline Nine-Hole Peg Test time above 22.15 seconds).

^d^Mann-Whitney *U* test with false discovery rate correction adjusted for age and sex.

^e^AUC: area under the curve.

**Table 5:** Ability of the fatigability features (aggregated by SD) to differentiate and distinguish between HCs^a^ and people with MS^b^ subgroups^c^.

Feature	HCs vs people with MS-Normal			HCs vs people with MS-Abnormal			People with MS-Normal vs people with MS-Abnormal		
	*P* value^d^	AUC^e^	Cohen *d*	*P* value^d^	AUC	Cohen *d*	*P* value^d^	AUC	Cohen *d*
Fatigability number of performed pinches	.87	0.52	0.00	.55	0.59	0.39	.43	0.59	0.39
Fatigability number of successful pinches	.94	0.51	0.08	.72	0.55	0.18	.74	0.54	0.07
Fatigability successful attempts fraction	.72	0.55	0.32	*.03* ^f^	*0.74*	*0.63*	*<.001*	*0.77*	*1.10*
Fatigability two-finger attempts fraction	.94	0.51	0.03	*.03*	*0.74*	*0.64*	*.01*	*0.75*	*0.71*
Fatigability pinch time	.72	0.55	0.40	.23	0.65	0.24	*.03*	*0.71*	*0.37*
Fatigability gap time	.78	0.54	0.06	.32	0.63	0.49	.16	0.64	0.46
Fatigability double touch asynchrony	.94	0.51	0.31	*.02*	*0.76*	*0.52*	*<.001*	*0.82*	*0.76*
Fatigability double lift asynchrony	.89	0.52	0.33	.20	0.66	0.21	.07	0.67	0.28
Fatigability first points distance	.72	0.56	0.18	.67	0.57	0.33	.18	0.64	0.52
Fatigability last points distance	.72	0.55	0.49	.63	0.58	0.15	.20	0.63	0.57
Fatigability finger path length	.72	0.55	0.43	.52	0.60	0.36	*.03*	*0.70*	*0.75*
Fatigability finger velocity	.87	0.52	0.24	.55	0.59	0.40	.67	0.56	0.22
Fatigability finger path ratio	.62	0.58	0.43	.51	0.60	0.32	.06	0.68	0.47

^a^HC: healthy control.

^b^MS: multiple sclerosis.

^c^This analysis included 18 HCs, 38 people with MS–Normal (ie, with a baseline Nine-Hole Peg Test time below 22.15 seconds), and 29 people with MS-Abnormal (ie, with a baseline Nine-Hole Peg Test time above 22.15 seconds).

^d^Mann-Whitney *U* test with false discovery rate correction adjusted for age and sex.

^e^AUC: area under the curve.

^f^Italic formatting represents a statistically significant *P* value at <.05.

### Relationship Between Pinching Test Features

To determine the common within-individual association for the assessed features, we conducted a repeated-measures correlation analysis [27]. The resulting correlation matrix showed only few correlations, suggesting that the different Pinching Test features captured different aspects of upper extremity function (Figure S6A in Multimedia Appendix 1). As expected, the number of performed pinches correlated with pinch time (correlation coefficient [CC]=–0.59), gap time (CC=–0.57), and finger velocity (CC=0.60), as slower pinching or larger gap time will lead to fewer pinches in a 30-second window.

The notion that most features capture unique information was also supported by the principal component analysis. While 4 principal components explained ~80% of the variance, 6 principal components explained 90% of it (Figure S6B in Multimedia Appendix 1). The factor analysis also revealed that the factors needed to explain the data captured by the Pinching Test features have distinct weights (Figure S6C in Multimedia Appendix 1).

## Discussion

### Principal Results

Our findings show that reliable features from the simple and ecologically valid smartphone sensor–based Pinching Test were associated with standard clinical measures of upper extremity function, overall disease severity, cognitive function, and fatigue; and identified people with MS with upper extremity function impairment. These features may also capture unique characteristics of limb movement not represented in standard clinical assessments. These data support the use of the Pinching Test to measure the ability to perform daily life activities (eg, grasping objects, buttoning a shirt, or controlling cutlery) and can be independently performed by patients. Furthermore, they also support the use of smartphone-based sensors to assess multiple aspects of pinching including the accuracy, efficiency, and smoothness of pinching, together with the range of motion and coordination of 2 fingers.

Ideal Pinching Test features generally fulfill three key criteria: (1) test-retest reliability, (2) agreement with standard clinical measures of the overall disease severity and upper extremity function (eg, the 9HPT, EDSS, and MSIS-29 arm items), and (3) ability to differentiate and distinguish between people with MS with and those without upper extremity functional impairment. Herein, we identified features fulfilling all 3 criteria, including most of the pinching features, such as number of performed pinches, number of successful pinches, two-finger attempts fraction, pinch time, gap time, double touch asynchrony, last points distance, and finger path ratio (Table S6 in Multimedia Appendix 1). These features demonstrated ICC values indicating moderate or good test-retest reliability, which is in line with previous studies on smartphone sensor–based assessments of upper extremity function in MS [36] and Parkinson disease [37]. However, most other features showed ICC values below the 0.84 previously reported for the 9HPT [38,39]. This is expected, considering the previously reported lower ICC values for sensor-based assessments compared with clinical measures [39]. Possible explanations include the lack of supervision, if administered remotely like the Pinching Test, and frequent administration, making them more susceptible to differences in test execution, motivation, and other confounders [40].

Agreement with both the clinician-reported assessments (9HPT time and EDSS) and patients’ perspectives of the impact of the disease (arm-related items of the MSIS-29) was strongest for pinching features, with a few exceptions. Successful attempts fraction, double lift asynchrony, and first points distance did not correlate with the standard clinical assessments for upper extremity function and overall disease severity measures; finger path length only correlated with 9HPT time, but not with EDSS or MSIS-29 arm items. However, successful attempts fraction, double lift asynchrony, and first points distance may be capturing functional abnormality not captured by the 9HPT. The overall mild levels of functional impairment (mean baseline 9HPT time of 22.1 seconds for the dominant hand; mean baseline EDSS 2.4) of the enrolled people with MS likely weakened the agreement between the Pinching Test features and these clinical measures.

Pinching as many tomato shapes as possible within 30 seconds on a smartphone device requires motor skills, coordination, fast information processing, and attention; it was thus expected that many pinching features correlated with both the 9HPT and oral SDMT. Additionally, overall disease severity confounds the association between these clinical measures, and thus some degree of correlation with the oral SDMT can be expected. Consequently, features that primarily capture a motor component would retain a significant correlation with 9HPT time even after accounting for the oral SDMT, while the correlation with the oral SDMT would tend to zero after accounting for 9HPT time. This was observed with the number of performed pinches, double touch asynchrony, finger path ratio, and gap time. Unsurprisingly, double touch asynchrony was found to be primarily driven by the motor components, as it measures the duration between the thumb and the second or third finger touching the smartphone screen at the beginning of the pinching gesture. As such, it was designed to be independent from cognitive tasks involved in recognizing a new tomato shape appearing on the screen.

Pinching features also best differentiated between people with MS-Normal and people with MS-Abnormal, indicating that greater levels of functional impairment resulted in poorer performance on the Pinching Test. Additionally, 3 such features—two-finger attempts fraction, gap time, and double touch asynchrony—also differentiated between HCs and people with MS-Abnormal. A global 9HPT threshold, derived from the normative population of Erasmus et al [30], which had a similar age and sex distribution as our cohort of people with MS, was used to classify people with MS as either people with MS-Normal or people with MS-Abnormal. However, the small numbers of HCs and people with MS-Abnormal and the imbalances in age and sex between the groups limited differentiation of HCs from people with MS subgroups. To address this, trial participants could be classified as people with MS-Normal vs people with MS-Abnormal by their upper extremity function through applying adaptive thresholds based on their age and sex. However, methodology involving adaptive thresholds is better suited for larger, more diverse studies. Examples of studies that could use adaptive thresholds include CONSONANCE (ClinicalTrials.gov NCT03523858) and Floodlight MS–TONiC (ISRCTN Registry ISRCTN11088592). Our future work will also explore multivariate analyses that fully use the Pinching Test’s multidimensional feature space.

By comparison, IMU-based features assessing the function of the stabilizing hand generally fulfilled only the test-retest reliability criterion (Table S6 in Multimedia Appendix 1). Their ICC(2,1) results were comparable to those obtained with the pinching features, but their performance in terms of their agreement with clinical measures and their ability to differentiate between HCs and people with MS subgroups was poorer. Considering most people with MS enrolled here had relapsing-remitting disease with minimal confirmed disability, it is possible that the amplitude of movement abnormalities or motor deficits encountered herein were too minute for these features to capture disease-related signals; thus, their utility will need to be further characterized in ongoing studies of primary progressive MS such as the CONSONANCE study.

Finally, we investigated features that could hypothetically capture fatigability. These features compared the performance during the first half vs the second half of the test. Some fatigability features fulfilled 2 out of 3 criteria (fatigability pinch time fulfilled all 3 criteria), but only when aggregating individual tests by SD (Table S6 in Multimedia Appendix 1). The aforementioned unreliable ICC values achieved for the fatigability features regardless of the aggregation method were expected due to the short test duration and the variability associated with fatigue. The improved performance of the SD aggregation may reflect fluctuations in fatigue, as it is commonly observed in people with MS [26]. However, possibly the duration of a single 30-second test run did not suffice to actively fatigue the study participants. This may explain why most fatigability features did not correlate with FSMC after accounting for 9HPT time and did not differentiate between fatigued and unfatigued people with MS. Therefore, these features may mostly capture aspects of upper extremity function other than fatigability.

### Limitations

Our work has 2 methodological limitations. First, enrolled people with MS were mostly limited to relapsing-remitting MS with mild upper extremity impairment and overall disease disability, restricting the generalizability of the results and likely limiting the range of clinically relevant signals captured. Second, the study duration (24 weeks) prohibited us from assessing the Pinching Test’s ability to detect MS disease progression. The feasibility of using sensor-based assessments to monitor upper extremity function over time was recently demonstrated, showing a worsening in the performance of tapping the index finger against the thumb—a movement resembling that assessed by our Pinching Test—suggesting subtle progression uncaptured by EDSS [41]. Additionally, our work presents an analytical limitation. Namely, it is difficult to investigate the relationship between fatigue as captured by instruments (eg, FSMC) and fatigability features derived from the Pinching Test; this hinders adequate face and construct validation of the measurements.

### Conclusions

In conclusion, the Pinching Test offers an objective, self-administered assessment of upper extremity function, potentially complementing the standard clinical evaluation of MS. Here, we investigated a range of features and identified those most reliably measuring various aspects of the pinching motion, such as accuracy, efficiency, responsiveness, and smoothness of pinching; agreement with clinical measures of upper extremity function and overall disease severity; and ability to differentiate between people with MS with and without upper extremity functional impairment. Taken together with our previous findings that people with MS were highly adherent to the Floodlight PoC active tests (70%; 16.68 out of 24 weeks) and had a high satisfaction score (73.7/100), this work adds evidence supporting the application’s use in MS monitoring [21]. Please see Multimedia Appendix 1 for a video summary of the data described herein, which was orally presented at the 2022 meeting of the European Committee for Treatment and Research in Multiple Sclerosis (see Multimedia Appendix 2). We encourage further exploration and evaluation of the Pinching Test in MS (eg, through assessment of sensitivity to longitudinal change or ability to detect a treatment effect), as well as in other relevant conditions (eg, Parkinson disease, trauma, and stroke). We also recommend adjustments of the statistical analysis for the SD-aggregated endpoints in future works, by determining the optimal schedule and quantity of test attempts required for these analyses. Our ongoing and future work will focus on further characterizing the Pinching Test in a broader patient population with more advanced disease and will examine the test’s effectiveness in detecting MS disease progression.

## Appendix

Multimedia Appendix 1 Supplementary appendix.

Multimedia Appendix 2 Oral presentation at ECTRIMS 2022.

## Abbreviations

9HPT: Nine-Hole Peg Test
AUC: area under the curve
CC: correlation coefficient
EDSS: Expanded Disability Status Scale
fs: sampling frequency
FSMC: Fatigue Scale for Motor and Cognitive Functions
FSS: functional system score
HC: healthy control
ICC: intraclass correlation coefficients
ICC(2,1): intraclass correlation coefficients (second model, first type)
IMU: inertial measurement unit
MS: multiple sclerosis
MSIS-29: 29-item Multiple Sclerosis Impact Scale
PoC: Proof-of-Concept
QoL: quality of life
SDMT: Symbol Digit Modalities Test

## Data Availability

For up-to-date details on Roche’s Global Policy on Sharing of Clinical Study Information and how to request access to related clinical study documents, see the website [42]. Request for the data underlying this publication requires a detailed, hypothesis-driven statistical analysis plan that is collaboratively developed by the requestor and company subject matter experts. Such requests should be directed to dbm.datarequest@roche.com for consideration. Anonymized records for individual patients across more than 1 data source external to Roche cannot, and should not, be linked due to a potential increase in the risk of patient reidentification.
