# Supplementary material for: Assessment of Upper Extremity Function in Multiple Sclerosis: Feasibility of a Digital Pinching Test
Source: JMIR Form Res. 2023 Oct 2;7:e46521. doi: 10.2196/46521 (PMC10580133; doi:10.2196/46521)
Supplement: Multimedia Appendix 1 [file formative_v7i1e46521_app1.pdf]

Supplementary appendix

Assessment of Upper Extremity Function in Multiple Sclerosis: Feasibility of a Digital Pinching Test

Jennifer S Graves[1], MD, PhD; Marcin Elantkowski[2], BSc; Yan-Ping Zhang[2], PhD; Frank Dondelinger[2]*, PhD; Florian Lipsmeier[2], PhD; Corrado Bernasconi[2], MD, PhD; Xavier Montalban[3], MD; Luciana Midaglia[3,4], MD; Michael Lindemann[2], PhD

[1]Department of Neurosciences, University of California, San Diego, California, United States

[2]F. Hoffmann-La Roche Ltd, Basel, Switzerland

[3]Department of Neurology-Neuroimmunology, Centre d'Esclerosi Múltiple de Catalunya (Cemcat), Hospital Universitari Vall d'Hebron, Barcelona, Spain

[4]Department of Medicine, Autonomous University of Barcelona, Barcelona, Spain

*at the time of writing author was an employee of F. Hoffmann-La Roche Ltd; current affiliation is Novartis Institutes for Biomedical Research, Basel, Switzerland

Figures

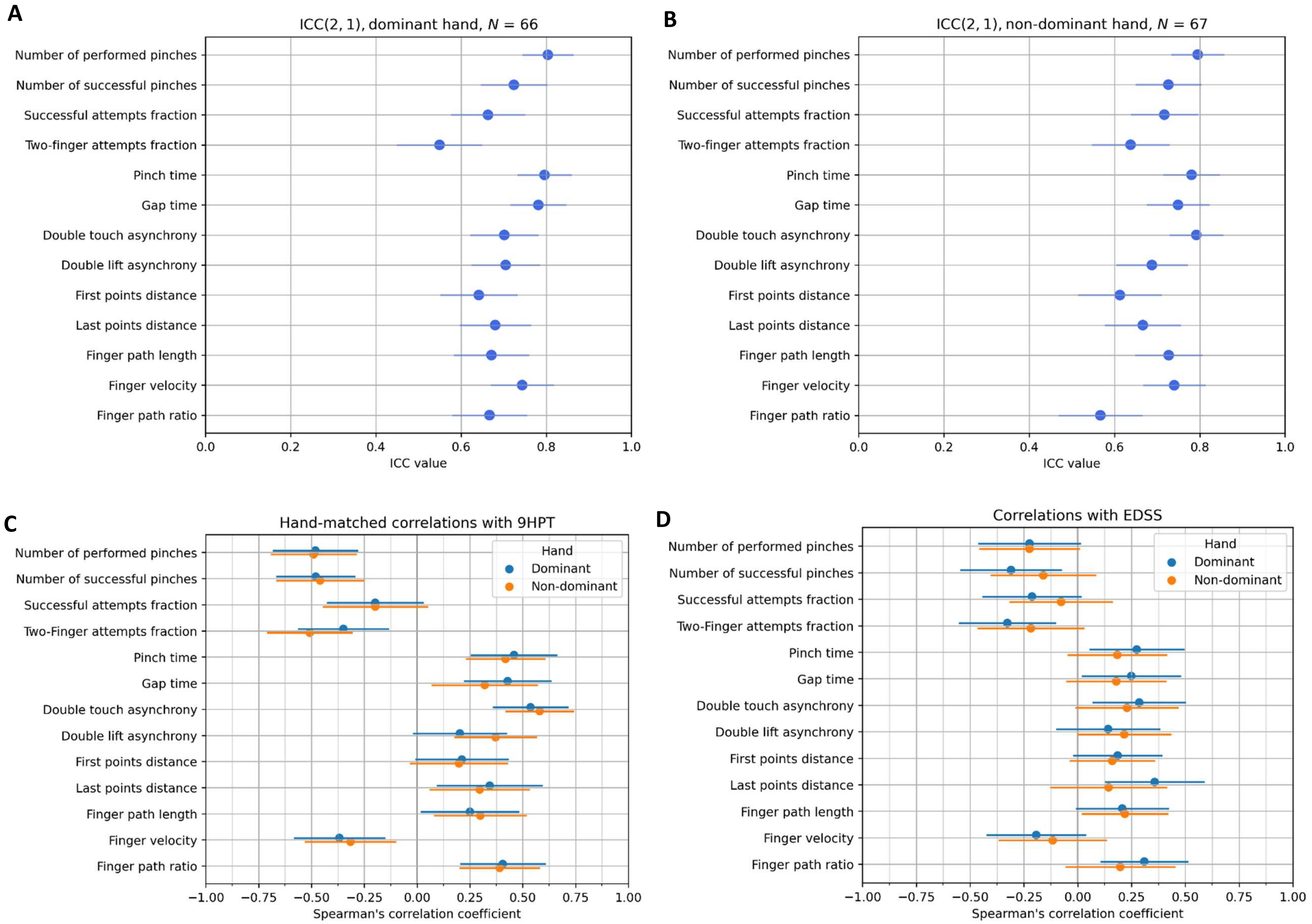

A
ICC(2, 1), dominant hand, N = 66
B
ICC(2, 1), non-dominant hand, N = 67
Number of performed pinches
Number of successful pinches
Successful attempts fraction
Two-finger attempts fraction
Pinch time
Gap time
Double touch asynchrony
Double lift asynchrony
First points distance
Last points distance
Finger path length
Finger velocity
Finger path ratio
0.0 0.2 0.4 0.6 0.8 1.0
ICC value
C
Hand-matched correlations with 9HPT
D
Correlations with EDSS
Two-Finger attempts fraction
Hand
Dominant
Non-dominant
−1.00 −0.75 −0.50 −0.25 0.00 0.25 0.50 0.75 1.00
Spearman's correlation coefficient

Figure S1. Test–retest reliability and correlations with 9HPT and EDSS of the Pinching Test's pinching features in PwMS for the dominant and nondominant hand. ICC(2,1) indicate comparable test–retest reliability for the (A) dominant hand and (B) nondominant hand. Age- and sex-corrected cross-sectional Spearman's rank correlations with (C) 9HPT and (D) EDSS were similar for the dominant hand (blue) and nondominant hand (orange). Error bars indicate 95% confidence interval estimated by bootstrapping. 9HPT: Nine-Hole Peg Test; EDSS: Expanded Disability Status Scale; ICC: intraclass correlation coefficients; PwMS: people with multiple sclerosis.

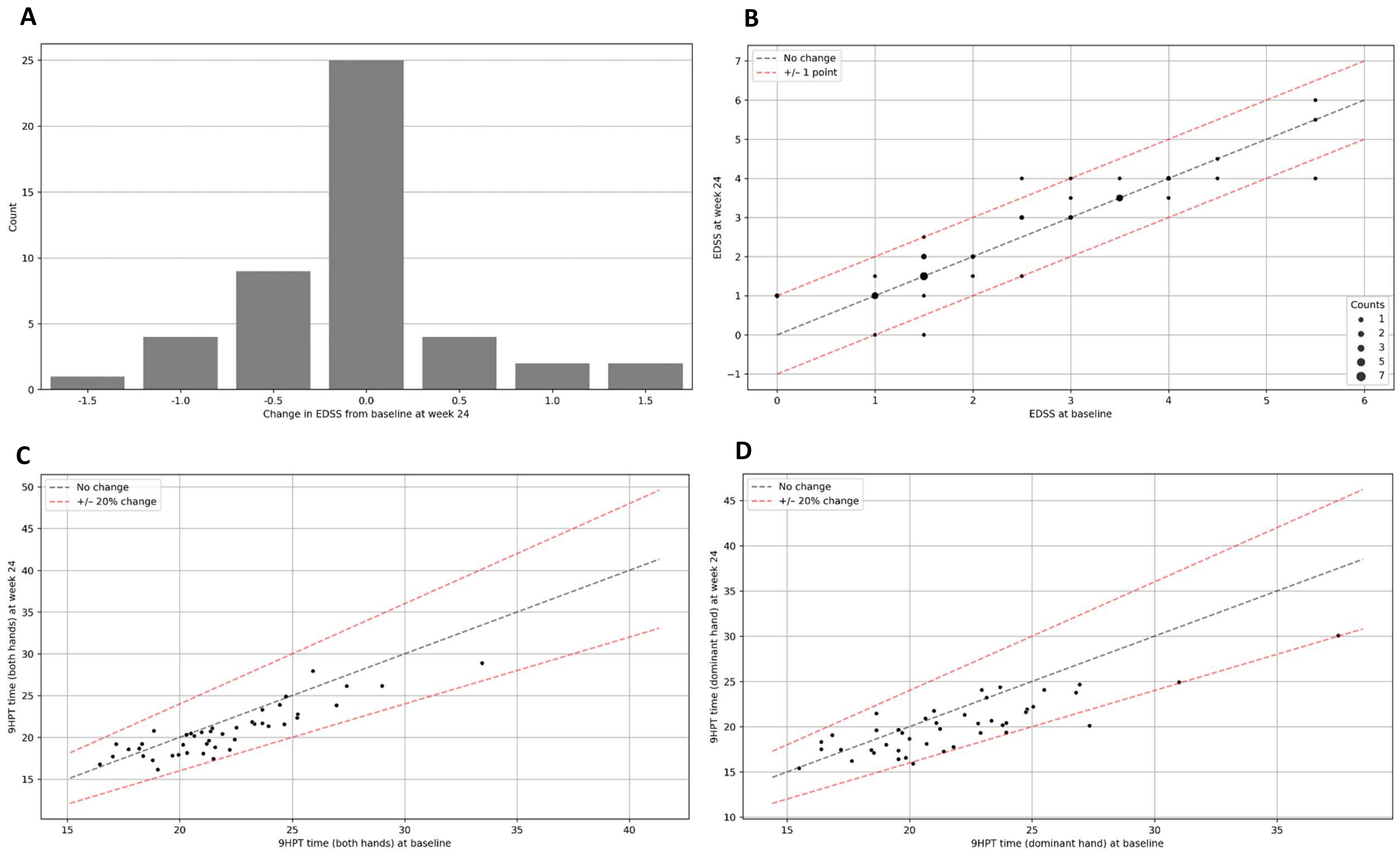

Figure S2. EDSS scores and 9HPT time at baseline and week 24 in PwMS. EDSS scores and 9HPT times were stable throughout the study period. (A) Histogram depicting the change in EDSS from baseline to week 24 (end of study). (B) Scatter plot depicting the EDSS scores at baseline and week 24. For most PwMS (number of PwMS indicated by the size of the “Counts” dot), the change was within 1 point on the EDSS scale (indicated by the red

dotted lines). (C-D) Scatter plots depicting the 9HPT times for (C) both hands combined and (D) dominant hand at baseline and week 24. For most PwMS, the change in 9HPT time was <20%. 9HPT: Nine-Hole Peg Test; EDSS: Expanded Disability Status Scale; PwMS: people with multiple sclerosis.

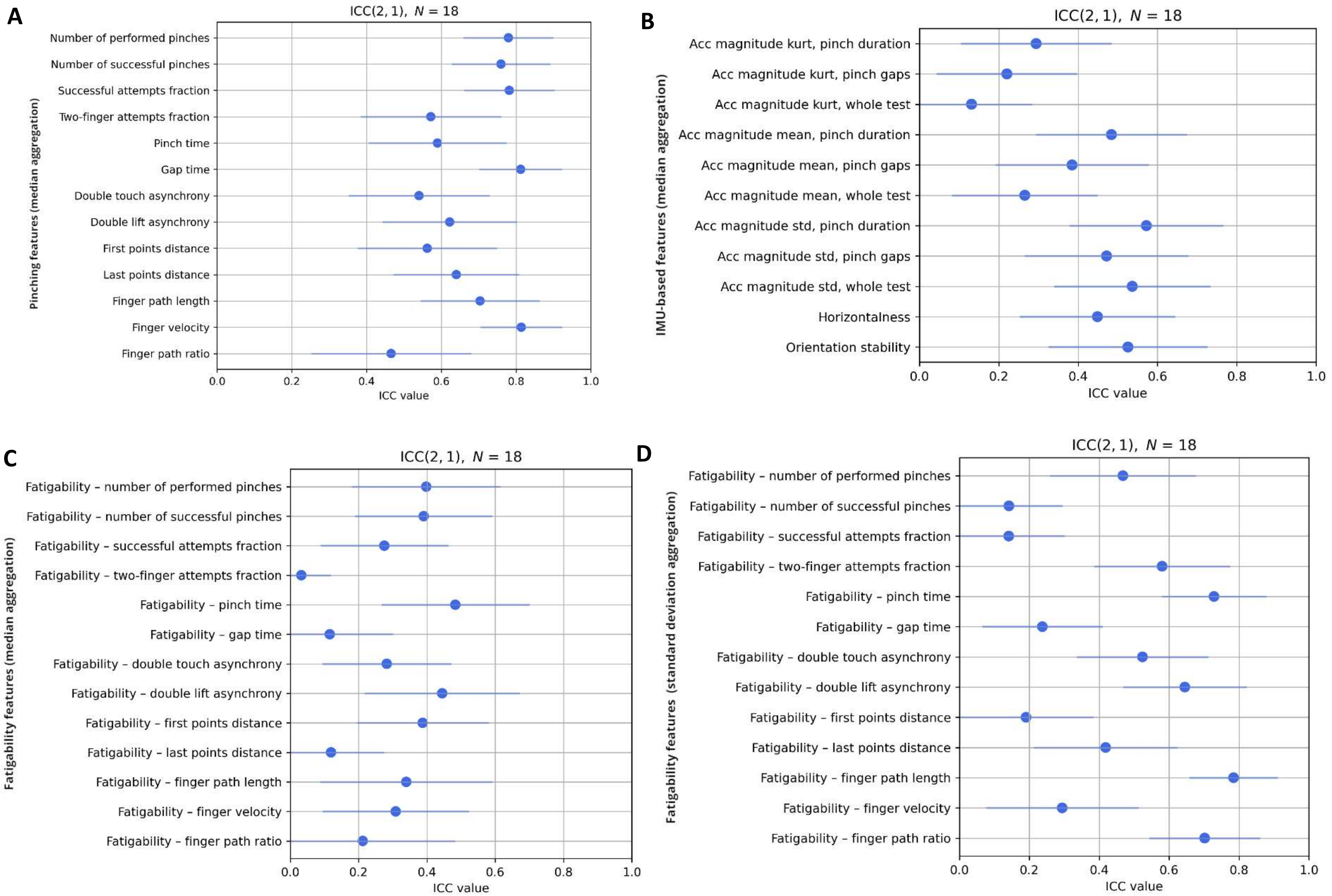
A
ICC(2, 1), N = 18
Number of performed pinches
Number of successful pinches
Successful attempts fraction
Two-finger attempts fraction
Pinch time
Gap time
Double touch asynchrony
Double lift asynchrony
First points distance
Last points distance
Finger path length
Finger velocity
Finger path ratio
Pinching features (median aggregation)
0.0 0.2 0.4 0.6 0.8 1.0
ICC value
B
ICC(2, 1), N = 18
Acc magnitude kurt, pinch duration
Acc magnitude kurt, pinch gaps
Acc magnitude kurt, whole test
Acc magnitude mean, pinch duration
Acc magnitude mean, pinch gaps
Acc magnitude mean, whole test
Acc magnitude std, pinch duration
Acc magnitude std, pinch gaps
Acc magnitude std, whole test
Horizontalness
Orientation stability
IMU-based features (median aggregation)
0.0 0.2 0.4 0.6 0.8 1.0
ICC value
C
ICC(2, 1), N = 18
Fatigability – number of performed pinches
Fatigability – number of successful pinches
Fatigability – successful attempts fraction
Fatigability – two-finger attempts fraction
Fatigability – pinch time
Fatigability – gap time
Fatigability – double touch asynchrony
Fatigability – double lift asynchrony
Fatigability – first points distance
Fatigability – last points distance
Fatigability – finger path length
Fatigability – finger velocity
Fatigability – finger path ratio
Fatigability features (median aggregation)
0.0 0.2 0.4 0.6 0.8 1.0
ICC value
D
ICC(2, 1), N = 18
Fatigability – number of performed pinches
Fatigability – number of successful pinches
Fatigability – successful attempts fraction
Fatigability – two-finger attempts fraction
Fatigability – pinch time
Fatigability – gap time
Fatigability – double touch asynchrony
Fatigability – double lift asynchrony
Fatigability – first points distance
Fatigability – last points distance
Fatigability – finger path length
Fatigability – finger velocity
Fatigability – finger path ratio
Fatigability features (standard deviation aggregation)
0.0 0.2 0.4 0.6 0.8 1.0
ICC value

Figure S3. Test–retest reliability in healthy controls. ICC(2,1) values of (A) pinching, (B) IMU-based, and (C-D) fatigability features. All consecutive 2-week windows with at least 3 valid test runs (per study participant) were included in the analyses. Feature values were aggregated across the 2-week windows by taking the (A-C) median or (D) standard deviation. Error bars indicate the 95% confidence interval estimated by bootstrapping. Acc: accelerometer; ICC: intraclass correlation coefficients; IMU: inertial measurement unit; kurt: kurtosis; std: standard deviation.

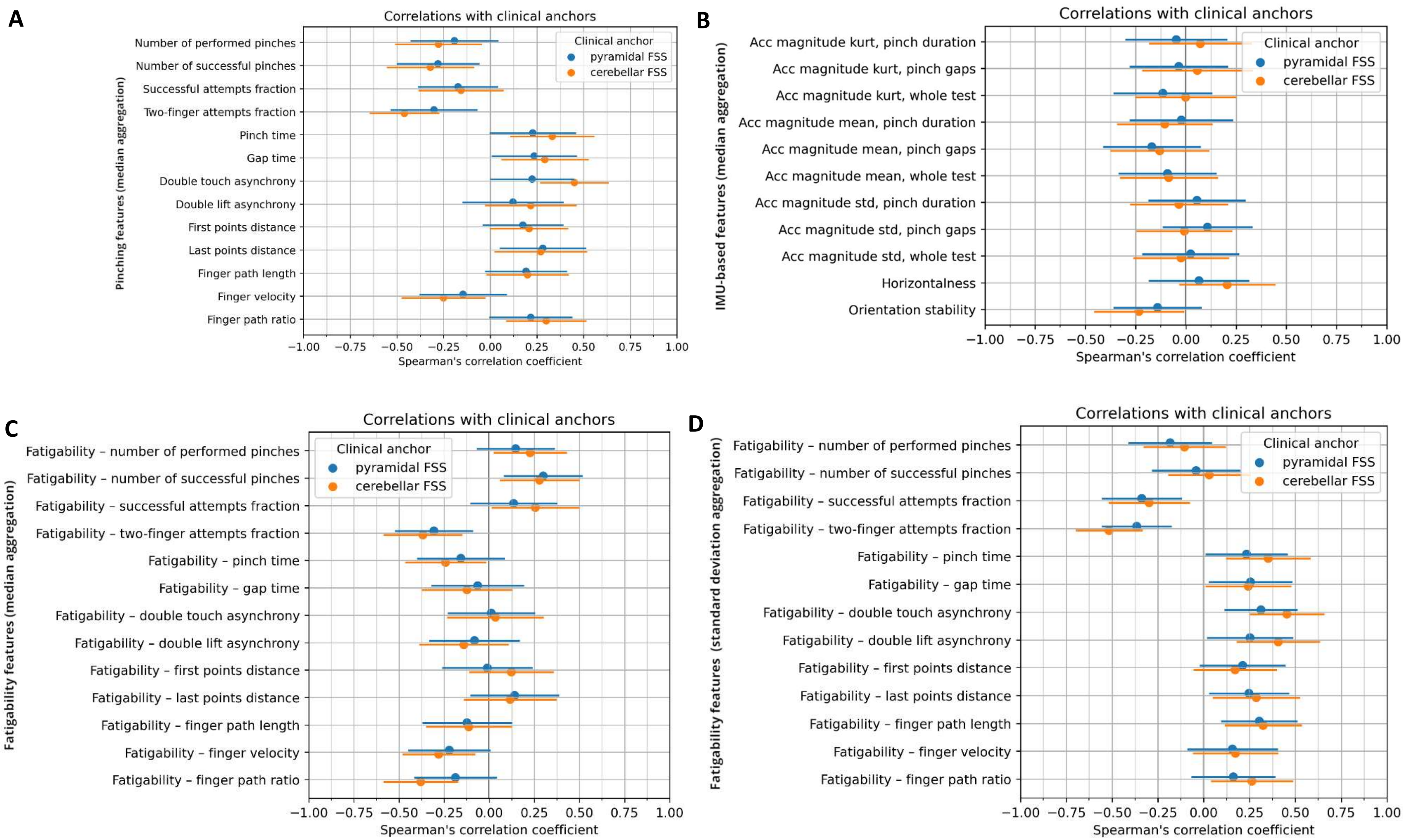

Figure S4. Cross-sectional Spearman's rank correlations between Pinching Test features and cerebellar as well as pyramidal FSS in PwMS. (A) pinching, (B) IMU-based, and (C-D) fatigability features were correlated against cerebellar and pyramidal FSSs after adjusting for age and sex. Error

bars indicate the 95% confidence interval estimated by bootstrapping. Acc: accelerometer; FSS: functional system score; IMU: inertial measurement unit; kurt: kurtosis; PwMS: people with multiple sclerosis; std: standard deviation.

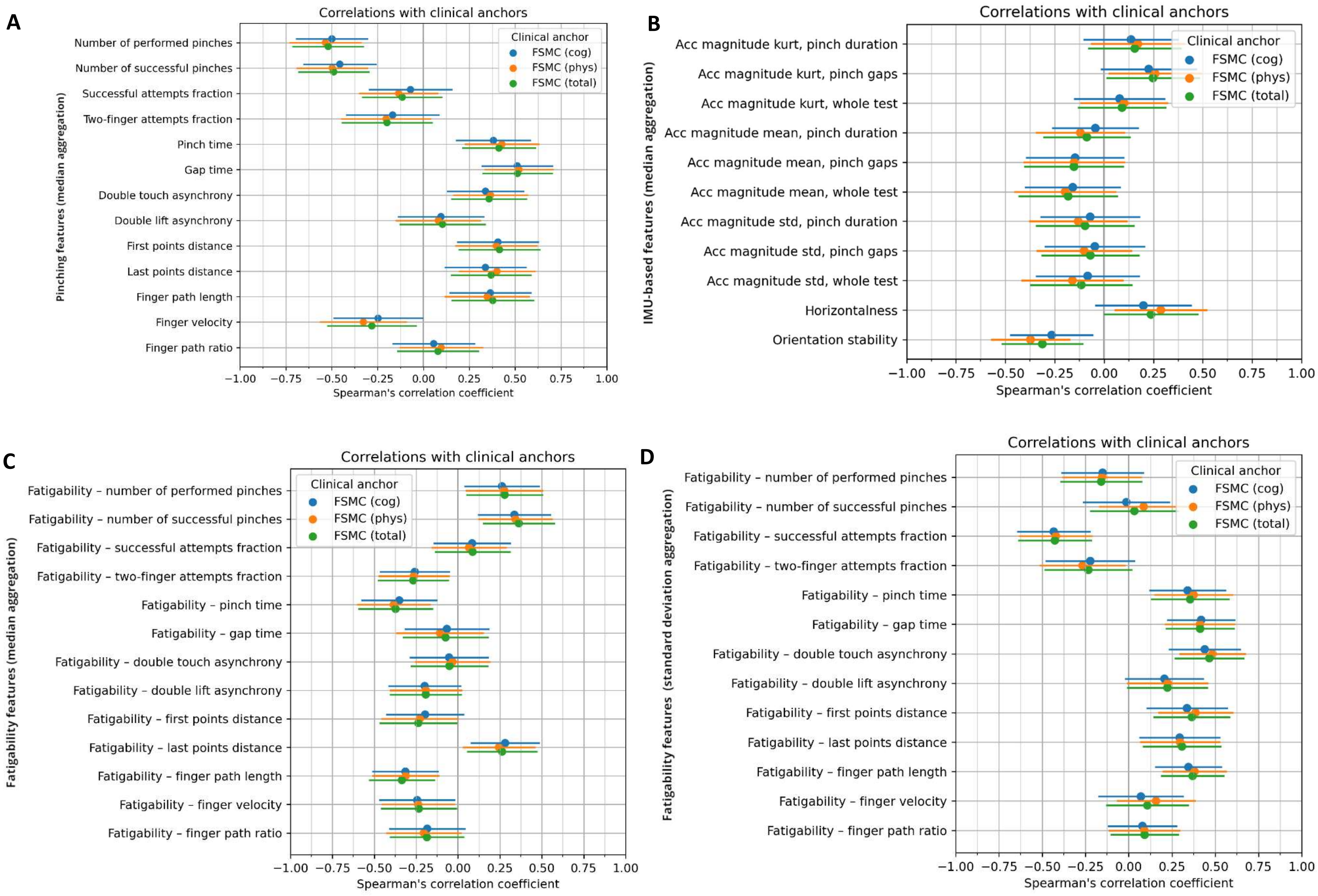

A
Correlations with clinical anchors
Pinching features (median aggregation)
Number of performed pinches
Number of successful pinches
Successful attempts fraction
Two-finger attempts fraction
Pinch time
Gap time
Double touch asynchrony
Double lift asynchrony
First points distance
Last points distance
Finger path length
Finger velocity
Finger path ratio
Clinical anchor
FSMC (cog)
FSMC (phys)
FSMC (total)
−1.00 −0.75 −0.50 −0.25 0.00 0.25 0.50 0.75 1.00
Spearman's correlation coefficient
B
Correlations with clinical anchors
IMU-based features (median aggregation)
Acc magnitude kurt, pinch duration
Acc magnitude kurt, pinch gaps
Acc magnitude kurt, whole test
Acc magnitude mean, pinch duration
Acc magnitude mean, pinch gaps
Acc magnitude mean, whole test
Acc magnitude std, pinch duration
Acc magnitude std, pinch gaps
Acc magnitude std, whole test
Horizontalness
Orientation stability
Clinical anchor
FSMC (cog)
FSMC (phys)
FSMC (total)
−1.00 −0.75 −0.50 −0.25 0.00 0.25 0.50 0.75 1.00
Spearman's correlation coefficient
C
Correlations with clinical anchors
Fatigability features (median aggregation)
Fatigability – number of performed pinches
Fatigability – number of successful pinches
Fatigability – successful attempts fraction
Fatigability – two-finger attempts fraction
Fatigability – pinch time
Fatigability – gap time
Fatigability – double touch asynchrony
Fatigability – double lift asynchrony
Fatigability – first points distance
Fatigability – last points distance
Fatigability – finger path length
Fatigability – finger velocity
Fatigability – finger path ratio
Clinical anchor
FSMC (cog)
FSMC (phys)
FSMC (total)
−1.00 −0.75 −0.50 −0.25 0.00 0.25 0.50 0.75 1.00
Spearman's correlation coefficient
D
Correlations with clinical anchors
Fatigability features (standard deviation aggregation)
Fatigability – number of performed pinches
Fatigability – number of successful pinches
Fatigability – successful attempts fraction
Fatigability – two-finger attempts fraction
Fatigability – pinch time
Fatigability – gap time
Fatigability – double touch asynchrony
Fatigability – double lift asynchrony
Fatigability – first points distance
Fatigability – last points distance
Fatigability – finger path length
Fatigability – finger velocity
Fatigability – finger path ratio
Clinical anchor
FSMC (cog)
FSMC (phys)
FSMC (total)
−1.00 −0.75 −0.50 −0.25 0.00 0.25 0.50 0.75 1.00
Spearman's correlation coefficient

Figure S5. Cross-sectional Spearman's rank correlations between Pinching Test features and physical and cognitive fatigue in PwMS. (A) pinching, (B) IMU-based, and (C-D) fatigability features were correlated against FSMC cognitive subscale (blue), FSMC physical subscale (orange), and FSMC total score (green) after adjusting for age and sex. Error bars indicate the 95% confidence interval estimated by bootstrapping. Acc: accelerometer; cog: cognitive; FSMC: Fatigue Scale for Motor and Cognitive Functions; IMU: inertial measurement unit; kurt: kurtosis; phys: physical; PwMS: people with multiple sclerosis; std: standard deviation.

A

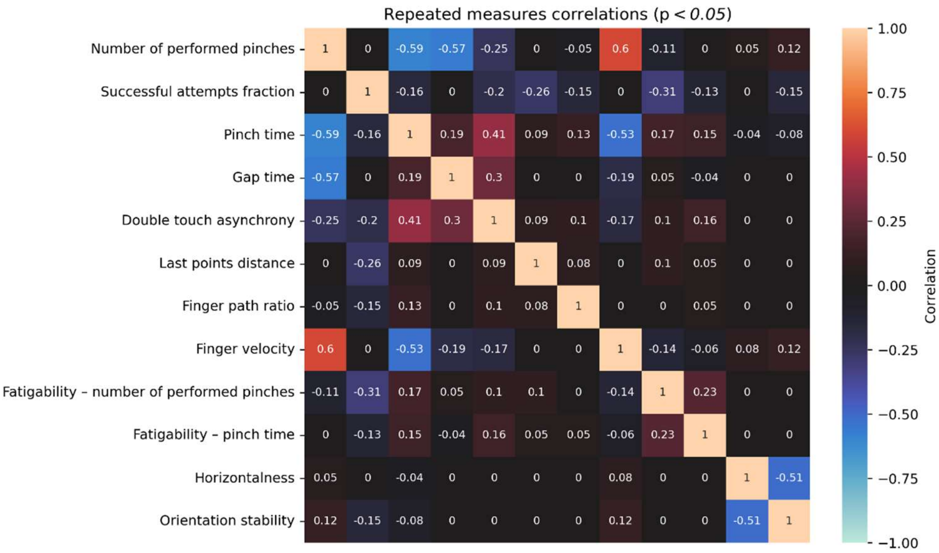

B

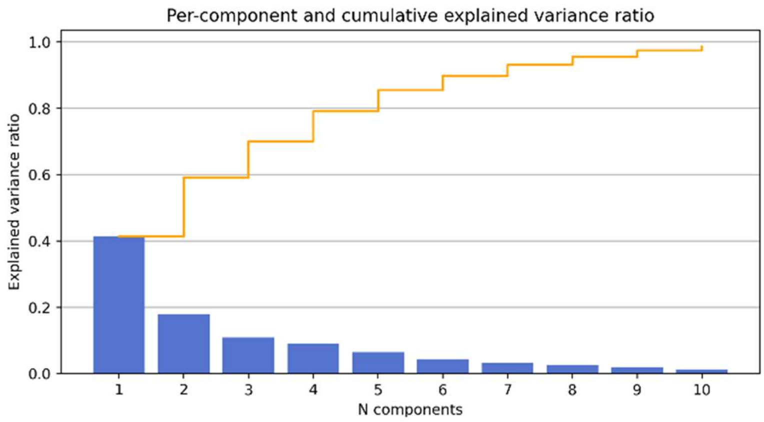

C

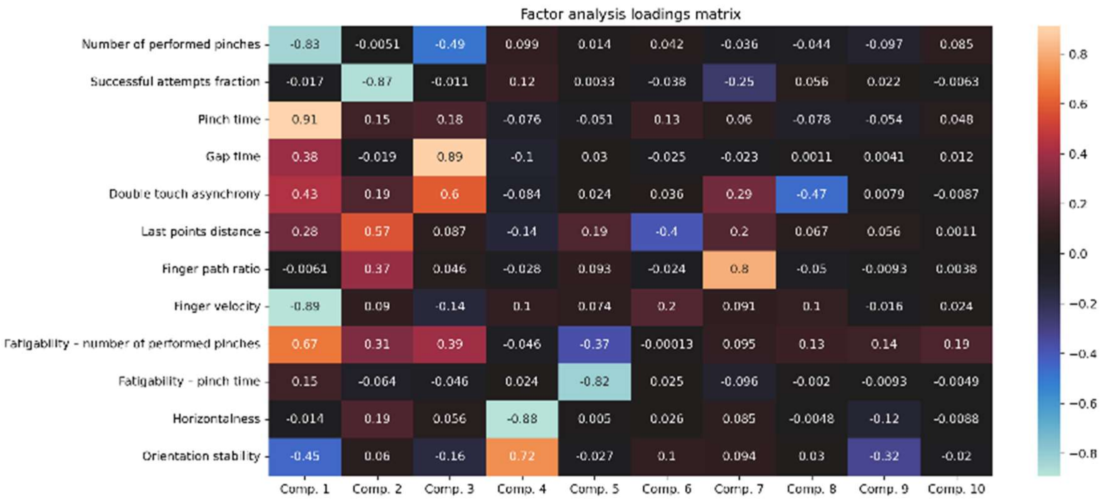

Figure S6. Relationship between the Pinching Test features. (A) Repeated-measures correlation analysis shows that the Pinching Test features within a single test run are not strongly correlated with each other.

(B) A principal component analysis revealed that 6 principal components are necessary to explain approximately 90% of the variance of the pinching features. (C) The loading matrix of the factor analysis further corroborates the notion that the individual pinching features all capture different aspects of upper extremity impairment. Comp.: Component.

Tables

Table S1. Definitions of Pinching Test features derived from the Pinching Test.

**Type**	**Definition**
Pinching features	
Number of (performed/ successful) pinches	Number of performed pinches = number of pinch attempts during the 30 secs of the test<br>Number of successful pinches = number of pinch attempts during the 30 secs of the test that resulted in a successfully pinched tomato shape
(Successful/two-finger) attempts fraction	$\text{Successful attempts fraction} = \frac{\text{Number of successful pinches}}{\text{Number of performed pinches}}$<br>$\text{Two-finger attempts fraction} = \frac{\text{Number of pinches performed with two fingers}}{\text{Number of performed pinches}}$
Double touch asynchrony	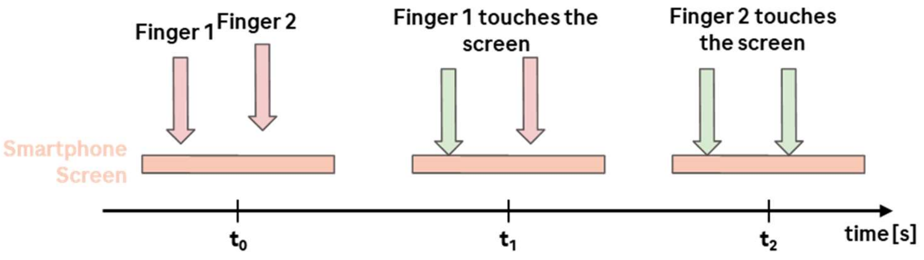<br>$\text{Double touch asynchrony} = t_2 - t_1$
Double lift asynchrony	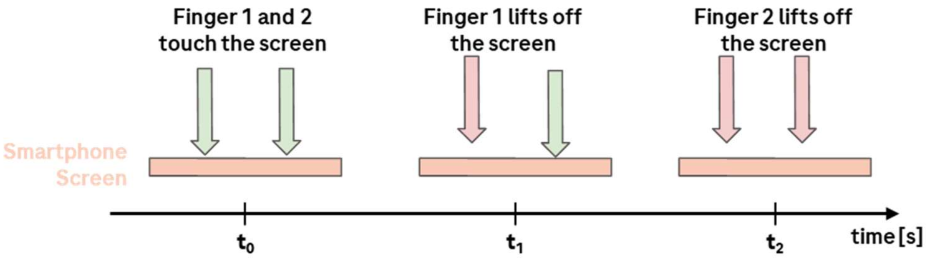<br>$\text{Double lift asynchrony} = t_2 - t_1$
Gap time	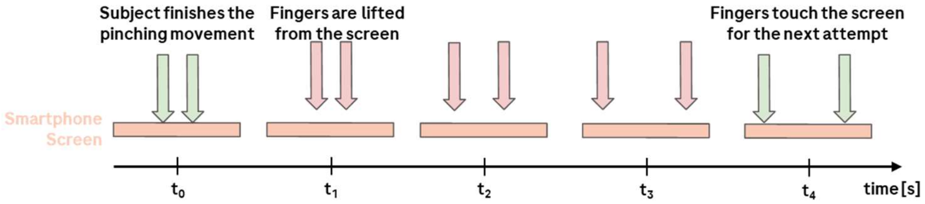<br>$\text{Gap time} = t_4 - t_0$

Pinch time

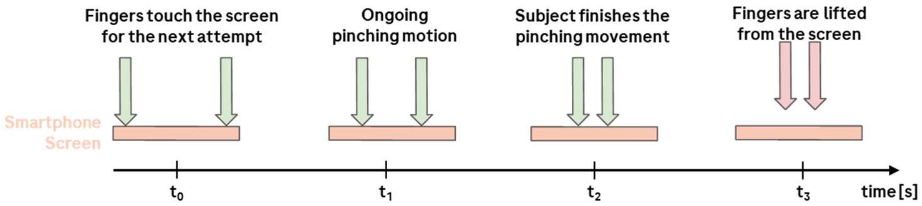

$$\text{Pinch time} = t_2 - t_0$$

Finger path length

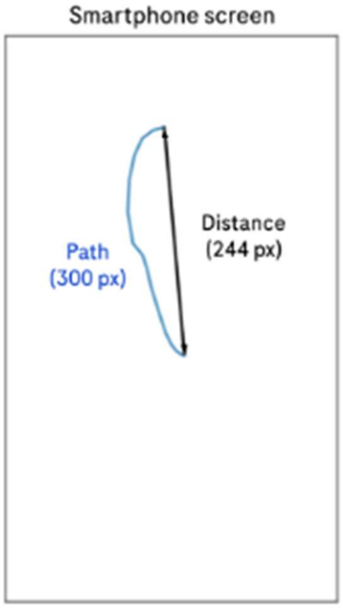

Finger path length: 300 px (blue line)

$$\text{Finger path ratio} = \frac{\text{Finger path length}}{\text{Distance between endpoints}}$$

$$\text{Finger path velocity} = \frac{\text{Finger path length}}{\text{Pinching duration}}$$

Distance between first points

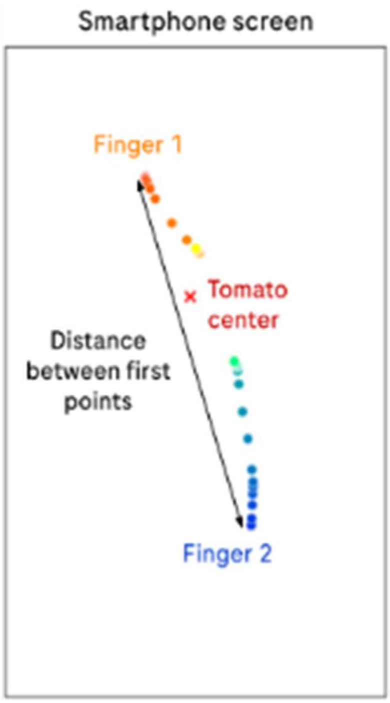

Distance between last points

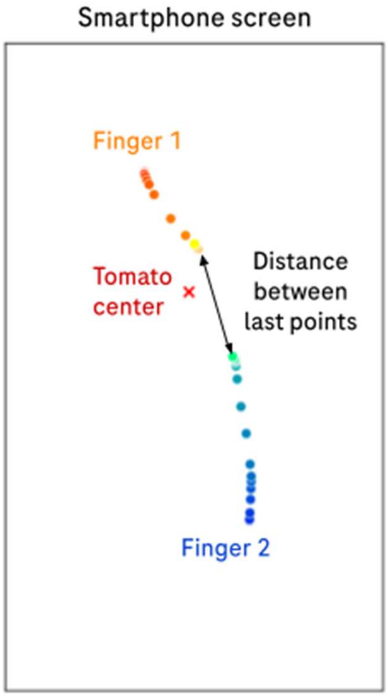

IMU-based features	
Mean accelerometer magnitude (entire test/pinch duration/pinch gap)	$\text{mean}\left(\sqrt{x^2+y^2+z^2}\right)$, where x, y, and z are the accelerometer signal in the x, y, and z axis, respectively. The accelerometer magnitude is computed for each time stamp and aggregated for either the entire test duration, during the pinching, or during pinch gaps.
SD accelerometer magnitude (entire test/pinch duration/pinch gap)	$\text{SD}\left(\sqrt{x^2+y^2+z^2}\right)$, where x, y, and z are the accelerometer signal in the x, y, and z axis, respectively, and SD is the standard deviation. The accelerometer magnitude is computed for each time stamp and aggregated for either the entire test duration, during the pinching, or during pinch gaps.
Kurtosis accelerometer magnitude (entire test/pinch duration/pinch gap)	$\text{Kurtosis}\left(\sqrt{x^2+y^2+z^2}\right)$, where x, y, and z are the accelerometer signal in the x, y, and z axis, respectively. The accelerometer magnitude is computed for each time stamp and aggregated for either the entire test duration, during the pinching, or during pinch gaps.
Horizontalness	$\text{abs}\left(\text{mean}\left[\frac{z}{\sqrt{x^2+y^2+z^2}}\right]\right)$, where x, y, and z are the accelerometer signal in the x, y, and z axis, respectively, computed for each time stamp.
Orientation stability	$\text{SD}\left(\frac{z}{\sqrt{x^2+y^2+z^2}}\right)$, where x, y, and z are the accelerometer signal in the x, y, and z axis, respectively, computed for each time stamp, and SD is the standard deviation.
Fatigability features	
Fatigability number of (performed/successful) pinches	Pinching feature value$_{t15–30}$ – pinching feature value$_{t0–15}$, where t0–15 refers to the first 15 seconds of the Pinching Test, t15–63 to the last 15 seconds of the Pinching Test.
Fatigability (successful/two-	Pinching feature value$_{t15–30}$ – pinching feature value$_{t0–15}$, where t0–15 refers to the first 15 seconds of the

finger) attempts fraction	Pinching Test, t15–63 to the last 15 seconds of the Pinching Test.
Fatigability double touch asynchrony	$\text{Mean}(\text{double touch asynchrony}_{t15-30}) - \text{mean}(\text{double touch asynchrony}_{t0-15})$, where t0–15 refers to the first 15 seconds of the Pinching Test, t15–63 to the last 15 seconds of the Pinching Test.
Fatigability double lift asynchrony	$\text{Mean}(\text{double lift asynchrony}_{t15-30}) - \text{mean}(\text{double lift asynchrony}_{t0-15})$, where t0–15 refers to the first 15 seconds of the Pinching Test, t15–63 to the last 15 seconds of the Pinching Test.
Fatigability gap time	$\text{Mean}(\text{gap time}_{t15-30}) - \text{mean}(\text{gap time}_{t0-15})$, where t0–15 refers to the first 15 seconds of the Pinching Test, t15–63 to the last 15 seconds of the Pinching Test.
Fatigability pinch time	$\text{Mean}(\text{pinch time}_{t15-30}) - \text{mean}(\text{pinch time}_{t0-15})$, where t0–15 refers to the first 15 seconds of the Pinching Test, t15–63 to the last 15 seconds of the Pinching Test.
Fatigability finger path length	$\text{Mean}(\text{finger path length}_{t15-30}) - \text{mean}(\text{finger path length}_{t0-15})$, where t0–15 refers to the first 15 seconds of the Pinching Test, t15–63 to the last 15 seconds of the Pinching Test.
Fatigability finger path ratio	$\text{Mean}(\text{finger path ratio}_{t15-30}) - \text{mean}(\text{finger path ratio}_{t0-15})$, where t0–15 refers to the first 15 seconds of the Pinching Test, t15–63 to the last 15 seconds of the Pinching Test.
Fatigability finger path velocity	$\text{Mean}(\text{finger path velocity}_{t15-30}) - \text{mean}(\text{finger path velocity}_{t0-15})$, where t0–15 refers to the first 15 seconds of the Pinching Test, t15–63 to the last 15 seconds of the Pinching Test.
Fatigability distance between first points	$\text{Mean}(\text{distance between first points}_{t15-30}) - \text{mean}(\text{distance between first points}_{t0-15})$, where t0–15 refers to the first 15 seconds of the Pinching Test, t15–63 to the last 15 seconds of the Pinching Test.
Fatigability distance between last points	$\text{Mean}(\text{distance between last points}_{t15-30}) - \text{mean}(\text{distance between last points}_{t0-15})$, where t0–15 refers to the first 15 seconds of the Pinching Test, t15–63 to the last 15 seconds of the Pinching Test.

IMU: inertial measurement unit; SD: standard deviation.

Table S2. Ability of dominant- and nondominant-handed pinching features to differentiate between PwMS with no-to-minimal disability from PwMS with at least mild disability on the cerebellar functional system.[a]

Features	HC vs PwMS with no-to-minimal disability			HC vs PwMS with at least mild disability			PwMS with no-to-minimal disability vs PwMS with at least mild disability		
	***P*[b]**	**AUC**	***d***	***P*[b]**	**AUC**	***d***	***P*[b]**	**AUC**	***d***
Dominant hand									
Number of performed pinches	0.82	0.53	0.27	0.49	0.63	0.56	0.52	0.60	0.40
Number of successful pinches	0.82	0.54	0.05	0.34	0.67	0.50	0.34	0.64	0.49
Successful attempts fraction	0.89	0.52	0.05	0.76	0.56	0.20	0.59	0.60	0.26
Two-finger attempts fraction	0.38	0.63	0.11	0.14	0.76	0.31	0.16	0.68	0.55
Pinch time	0.91	0.51	0.08	0.82	0.54	0.32	0.64	0.58	0.21
Gap time	0.49	0.62	0.49	0.14	0.75	0.77	0.22	0.67	0.56
Double touch asynchrony	0.82	0.53	0.10	0.15	0.73	0.77	0.14	0.71	0.81
Double lift asynchrony	0.94	0.51	0.27	0.70	0.58	0.14	0.65	0.57	0.43
First points distance	0.82	0.53	0.02	0.65	0.59	0.29	0.64	0.59	0.25
Last points distance	0.64	0.58	0.23	0.70	0.58	0.22	0.28	0.65	0.47
Finger path length	0.76	0.55	0.41	0.82	0.53	0.23	0.64	0.58	0.26
Finger velocity	0.82	0.53	0.13	0.76	0.56	0.30	0.76	0.55	0.18
Finger path ratio	0.82	0.53	0.44	0.65	0.59	0.29	0.34	0.63	0.34
Nondominant hand									
Number of performed pinches	0.90	0.53	0.27	0.90	0.62	0.47	0.90	0.58	0.27
Number of successful pinches	0.90	0.54	0.13	0.90	0.60	0.35	0.90	0.56	0.24
Successful attempts fraction	0.90	0.52	0.09	0.90	0.53	0.12	0.90	0.52	0.03
Two-finger attempts fraction	0.90	0.52	0.35	0.90	0.55	0.07	0.90	0.58	0.52
Pinch time	0.95	0.50	0.10	0.90	0.53	0.21	0.90	0.51	0.11
Gap time	0.90	0.53	0.28	0.90	0.65	0.64	0.90	0.63	0.38
Double touch asynchrony	0.90	0.60	0.29	0.90	0.68	0.66	0.90	0.60	0.37
Double lift asynchrony	0.90	0.52	0.20	0.90	0.56	0.07	0.90	0.58	0.15

First points distance	0.90	0.55	0.09	0.90	0.55	0.18	0.90	0.59	0.27
Last points distance	0.90	0.53	0.00	0.90	0.54	0.12	0.90	0.55	0.11
Finger path length	0.90	0.58	0.37	0.90	0.55	0.15	0.90	0.62	0.28
Finger velocity	0.90	0.55	0.27	0.90	0.52	0.17	0.90	0.52	0.08
Finger path ratio	0.90	0.51	0.41	0.90	0.57	0.21	0.90	0.57	0.44

[a]This analysis included 18 HC, 48 PwMS with no-to-minimal disability on the cerebellar functional system (cerebellar FSS: ≤1), and 19 PwMS with at least mild disability on the cerebellar functional system (cerebellar FSS: ≥2).

[b]Mann–Whitney U test with FDR correction adjusted for age and sex.

AUC: area under the curve; FDR: false discovery rate; FSS: functional system score; HC: healthy controls; PwMS: people with multiple sclerosis.

Table S3. Ability of dominant- and nondominant-handed pinching features to differentiate between PwMS with no-to-minimal disability from PwMS with at least mild disability on the pyramidal functional system.[a]

**Features**	**HC vs PwMS with no-to-minimal disability**			**HC vs PwMS with at least mild disability**			**PwMS with no-to-minimal disability vs PwMS with at least mild disability**		
	***P*[b]**	**AUC**	***d***	***P*[b]**	**AUC**	***d***	***P*[b]**	**AUC**	***d***
Dominant hand									
Number of performed pinches	0.97	0.53	0.27	0.48	0.65	0.66	0.48	0.63	0.58
Number of successful pinches	0.86	0.56	0.11	0.48	0.65	0.46	0.82	0.58	0.41
Successful attempts fraction	0.97	0.51	0.02	0.97	0.52	0.01	0.97	0.51	0.02
Two-finger attempts fraction	0.48	0.66	0.02	0.48	0.69	0.06	0.87	0.55	0.06
Pinch time	0.97	0.52	0.01	0.79	0.61	0.61	0.48	0.65	0.61
Gap time	0.48	0.63	0.53	0.48	0.75	0.81	0.48	0.63	0.67
Double touch asynchrony	0.82	0.57	0.26	0.51	0.64	0.57	0.85	0.56	0.47
Double lift asynchrony	0.97	0.53	0.24	0.97	0.50	0.16	0.97	0.52	0.45
First points distance	0.97	0.52	0.04	0.82	0.59	0.50	0.72	0.60	0.58
Last points distance	0.82	0.56	0.23	0.87	0.57	0.31	0.48	0.64	0.58
Finger path length	0.95	0.54	0.42	0.97	0.52	0.19	0.82	0.57	0.33
Finger velocity	0.97	0.51	0.07	0.48	0.67	0.60	0.48	0.67	0.60
Finger path ratio	0.97	0.51	0.45	0.97	0.53	0.27	0.87	0.55	0.21
Nondominant hand									
Number of performed pinches	0.96	0.54	0.28	0.96	0.61	0.51	0.96	0.56	0.32
Number of successful pinches	0.96	0.56	0.19	0.96	0.54	0.19	0.96	0.52	0.01
Successful attempts fraction	0.96	0.54	0.17	0.96	0.54	0.19	0.96	0.59	0.34
Two-finger attempts fraction	0.96	0.51	0.26	0.96	0.54	0.24	0.96	0.51	0.14
Pinch time	0.96	0.52	0.03	0.96	0.60	0.46	0.96	0.59	0.47
Gap time	0.96	0.57	0.36	0.96	0.54	0.46	0.98	0.50	0.15
Double touch asynchrony	0.96	0.62	0.39	0.96	0.62	0.43	0.96	0.51	0.07
Double lift asynchrony	0.96	0.51	0.22	0.96	0.56	0.03	0.96	0.57	0.30

First points distance	0.96	0.55	0.13	0.96	0.60	0.44	0.96	0.65	0.56
Last points distance	0.96	0.51	0.02	0.96	0.51	0.06	0.96	0.51	0.04
Finger path length	0.96	0.57	0.35	0.96	0.54	0.17	0.96	0.62	0.20
Finger velocity	0.96	0.53	0.20	0.96	0.60	0.39	0.96	0.57	0.23
Finger path ratio	0.96	0.54	0.38	0.96	0.51	0.27	0.96	0.52	0.07

[a]This analysis included 18 HC, 53 PwMS with no-to-minimal disability on the pyramidal functional system (pyramidal FSS: ≤2), and 14 PwMS with at least mild disability on the pyramidal functional system (pyramidal FSS: ≥3).

[b]Mann–Whitney U test with FDR correction adjusted for age and sex.

AUC: area under the curve; FDR: false discovery rate; FSS: functional system score; HC: healthy controls; PwMS: people with multiple sclerosis.

Table S4. Ability of fatigability features (aggregated by median) to differentiate between PwMS without vs PwMS with at least mild levels of fatigue.[a]

Features	PwMS without vs PwMS with at least mild levels of fatigue		
	***P*[b]**	**AUC**	***d***
Fatigability number of performed pinches	0.29	0.62	0.48
Fatigability number of successful pinches	0.12	0.66	0.58
Fatigability successful attempts fraction	0.53	0.57	0.15
Fatigability two-finger attempts fraction	0.11	0.69	0.10
Fatigability pinch time	0.11	0.67	0.27
Fatigability gap time	0.86	0.52	0.07
Fatigability double touch asynchrony	0.91	0.51	0.09
Fatigability double lift asynchrony	0.11	0.66	0.20
Fatigability first points distance	0.53	0.57	0.34
Fatigability last points distance	0.53	0.57	0.30
Fatigability finger path length	0.11	0.67	0.56
Fatigability finger velocity	0.57	0.56	0.13
Fatigability finger path ratio	0.11	0.69	0.55

[a]This analysis included 17 PwMS without (FSMC total score <43) and 50 PwMS with at least mild levels of fatigue (FSMC total score ≥43).

[b]Mann–Whitney U test with FDR correction adjusted for age and sex.

AUC: area under the curve; FDR: false discovery rate; FSMC: Fatigue Scale for Motor and Cognitive Function; PwMS: people with multiple sclerosis.

Table S5. Ability of fatigability features (aggregated by SD) to differentiate between PwMS without vs PwMS with at least mild levels of fatigue.[a]

Features	PwMS without vs PwMS with at least mild levels of fatigue		
	***P*[b]**	**AUC**	***d***
Fatigability number of performed pinches	0.95	0.50	0.20
Fatigability number of successful pinches	0.41	0.58	0.14
Fatigability successful attempts fraction	0.20	0.65	0.57
Fatigability two-finger attempts fraction	0.22	0.62	0.47
Fatigability pinch time	0.20	0.65	0.50
Fatigability gap time	0.04	0.72	0.63
Fatigability double touch asynchrony	0.04	0.73	0.50
Fatigability double lift asynchrony	0.41	0.58	0.35
Fatigability first points distance	0.20	0.64	0.47
Fatigability last points distance	0.20	0.64	0.49
Fatigability finger path length	0.22	0.63	0.53
Fatigability finger velocity	0.23	0.61	0.39
Fatigability finger path ratio	0.74	0.53	0.33

[a]This analysis included 17 PwMS without (FSMC total score <43) and 50 PwMS with at least mild levels of fatigue (FSMC total score ≥43).

[b]Mann–Whitney U test with FDR correction adjusted for age and sex. Green background highlights a statistically significant *P* value at <.05.

AUC: area under the curve; FDR: false discovery rate; FSMC: Fatigue Scale for Motor and Cognitive Function; PwMS: people with multiple sclerosis; SD: standard deviation.

Table S6. Overview of test–retest reliability, agreement with standard clinical measures, and ability to differentiate and distinguish PwMS subgroups.

Feature	Test–retest reliability in PwMS	Correlations with standard clinical measures in PwMS[a]			Ability to differentiate between PwMS-Normal and PwMS-Abnormal[a,b]
		9HPT	EDSS	MSIS-29 arm items	
Pinching features (median aggregation)					
Number of performed pinches	Good	Fair	Uncorrelated	Fair	Yes
Number of successful pinches	Moderate	Fair	Fair	Fair	Yes
Successful attempts fraction	Moderate	Uncorrelated	Uncorrelated	Uncorrelated	No
Two-finger attempts fraction	Moderate	Fair	Fair	Uncorrelated	Yes
Pinch time	Good	Fair	Fair	Fair	Yes
Gap time	Good	Fair	Fair	Moderate-to-good	Yes
Double touch asynchrony	Moderate	Moderate-to-good	Fair	Fair	Yes
Double lift asynchrony	Moderate	Uncorrelated	Uncorrelated	Uncorrelated	No
First points distance	Moderate	Uncorrelated	Uncorrelated	Uncorrelated	No
Last points distance	Moderate	Fair	Fair	Fair	Yes
Finger path length	Moderate	Fair	Uncorrelated	Uncorrelated	No
Finger velocity	Moderate	Fair	Uncorrelated	Fair	No
Finger path ratio	Moderate	Fair	Fair	Uncorrelated	Yes

IMU-based features (median aggregation)					
Acc magnitude kurt, pinch duration	Moderate	Uncorrelated	Uncorrelated	Uncorrelated	No
Acc magnitude kurt, pinch gaps	Moderate	Uncorrelated	Uncorrelated	Uncorrelated	No
Acc magnitude kurt, whole test	Moderate	Uncorrelated	Uncorrelated	Uncorrelated	No
Acc magnitude mean, pinch duration	Moderate	Uncorrelated	Uncorrelated	Uncorrelated	No
Acc magnitude mean, pinch gaps	Moderate	Uncorrelated	Uncorrelated	Uncorrelated	No
Acc magnitude mean, whole test	Moderate	Uncorrelated	Uncorrelated	Uncorrelated	No
Acc magnitude SD, pinch duration	Good	Uncorrelated	Uncorrelated	Uncorrelated	No
Acc magnitude SD, pinch gaps	Good	Uncorrelated	Uncorrelated	Uncorrelated	No
Acc magnitude SD, whole test	Good	Uncorrelated	Uncorrelated	Uncorrelated	No
Horizontalness	Moderate	Uncorrelated	Uncorrelated	Fair	No
Orientation stability	Moderate	Fair	Uncorrelated	Fair	No
Fatigability features (median aggregation)					
Fatigability – number of performed pinches	Poor	Fair	Uncorrelated	Uncorrelated	No
Fatigability – number of successful pinches	Poor	Fair	Fair	Uncorrelated	No
Fatigability – successful attempts fraction	Poor	Uncorrelated	Uncorrelated	Uncorrelated	No
Fatigability – two-finger attempts fraction	Poor	Uncorrelated	Fair	Uncorrelated	No

Fatigability – pinch time	Poor	Uncorrelated	Fair	Fair	No
Fatigability – gap time	Moderate	Uncorrelated	Uncorrelated	Uncorrelated	No
Fatigability – double touch asynchrony	Poor	Uncorrelated	Uncorrelated	Uncorrelated	No
Fatigability – double lift asynchrony	Poor	Uncorrelated	Uncorrelated	Fair	No
Fatigability – first points distance	Poor	Uncorrelated	Uncorrelated	Uncorrelated	No
Fatigability – last points distance	Poor	Uncorrelated	Uncorrelated	Fair	No
Fatigability – finger path length	Poor	Uncorrelated	Uncorrelated	Fair	No
Fatigability – finger velocity	Poor	Uncorrelated	Fair	Uncorrelated	No
Fatigability – finger path ratio	Poor	Uncorrelated	Fair	Uncorrelated	No
Fatigability features (SD aggregation)					
Fatigability – number of performed pinches	Poor	Uncorrelated	Uncorrelated	Uncorrelated	No
Fatigability – number of successful pinches	Poor	Uncorrelated	Uncorrelated	Uncorrelated	No
Fatigability – successful attempts fraction	Poor	Fair	Fair	Fair	Yes
Fatigability – two-finger attempts fraction	Poor	Fair	Fair	Fair	Yes
Fatigability – pinch time	Moderate	Fair	Fair	Fair	Yes
Fatigability – gap time	Poor	Fair	Fair	Fair	No
Fatigability – double touch	Poor	Moderate-to-good	Fair	Fair	Yes

asynchrony					
Fatigability – double lift asynchrony	Poor	Fair	Fair	Uncorrelated	No
Fatigability – first points distance	Poor	Fair	Uncorrelated	Fair	No
Fatigability – last points distance	Poor	Fair	Fair	Uncorrelated	No
Fatigability – finger path length	Poor	Fair	Fair	Fair	Yes
Fatigability – finger velocity	Poor	Uncorrelated	Uncorrelated	Uncorrelated	No
Fatigability – finger path ratio	Poor	Fair	Uncorrelated	Uncorrelated	No

Green background highlights Pinching Test features that fulfill all 3 criteria (test–retest reliability [moderate or better], agreement with standard clinical measures [at least fair correlation with 2 or more of the clinical measures assessing either upper extremity function or overall disease severity, ie, 9HPT, EDSS, MSIS-29 arm items], and ability to differentiate between PwMS subgroups [*P*<.05]). Yellow background highlights features that fulfill 2 of these 3 criteria.

[a]Adjusted for age and sex.

[b]PwMS-Normal/-Abnormal: PwMS with a baseline 9HPT time below/above 22.15 seconds for the dominant hand, respectively.

9HPT: Nine-Hole Peg Test; acc: accelerometer; EDSS: Expanded Disability Status Scale; IMU: inertial measurement unit; kurt: kurtosis; MSIS-29: 29-item Multiple Sclerosis Impact Scale; PwMS: people with multiple sclerosis; SD: standard deviation.
